# Life’s Essential 8, Polygenic Risk for Type 2 Diabetes, and Dementia: Evidence from the UK Biobank

**DOI:** 10.3390/nu18132080

**Published:** 2026-06-25

**Authors:** Yuanjing Li, Örjan Ekblom, Bikram Bucha, Ariana Cojocari, Hui-Xin Wang, Rui Wang

**Affiliations:** 1 Department of Health Sciences, The Swedish School of Sport and Health Sciences, 11486 Stockholm, Sweden; orjan.ekblom@gih.se (Ö.E.); bikram.bucha@gih.se (B.B.); ariana.cojocari@gih.se (A.C.); 2 Division of Nursing, Department of Neurobiology, Care Sciences and Society, Karolinska Institutet, 14157 Huddinge, Sweden; 3 Master’s Programme in Nutrition Science, Karolinska Institutet, 14157 Huddinge, Sweden; 4 Stress Research Institute, Department of Psychology, Stockholm University, 11419 Stockholm, Sweden; huixin.wang@su.se; 5 Division of Clinical Geriatrics, Department of Neurobiology, Care Sciences and Society, Karolinska Institutet, 17164 Solna, Sweden; 6 Wisconsin Alzheimer’s Disease Research Center, School of Medicine and Public Health, University of Wisconsin, Madison, WI 53705, USA

**Keywords:** type 2 diabetes polygenic risk, dementia prevention, healthy lifestyle, metabolic dysfunction, UK Biobank cohort

## Abstract

Background/Objectives: It is unclear if adherence to healthy guidelines can modify the association between polygenic risk score (PRS) for type 2 diabetes and dementia. This study aimed to investigate interrelationships between PRS for type 2 diabetes, Life’s Essential 8 (LE8) metrics, and dementia. Methods: We included 437,732 UK Biobank participants aged ≥40 years between 2006 and 2010. PRS for type 2 diabetes was calculated by summing weighted genetic variant effects. Incident all-cause and cause-specific dementias were identified using registry records up to December 2022. LE8 scores were classified as low vs. moderate-to-high levels. Cox regression and restricted cubic splines were applied. Results: Over an average follow-up of 13.27 years (SD = 2.27), 9425 participants developed dementia. A dose–response relationship was observed between PRS and vascular dementia, with risk rising sharply beyond the 95th percentile. Individuals with low LE8 constantly showed a higher risk of all-cause dementia than those with moderate-to-high LE8 across all values of PRS for type 2 diabetes. *APOE* ε4 accounted for more than 35% of the population-attributable risk of dementia, whereas the PRS for type 2 diabetes contributed only 1%. The population-attributable risk of all-cause dementia could be further reduced by 5.91% to 10.46% through maintaining moderate-to-high LE8 behavioral components, even after considering *APOE* ε4. Conclusions: A dose–response relationship exists between PRS for type 2 diabetes and dementia, particularly vascular dementia. Adherence to optimal LE8 metrics, particularly behavioral components, may contribute to dementia prevention across genetic strata. These findings highlight the importance of multidomain lifestyle interventions in dementia prevention.

## 1. Introduction

Type 2 diabetes is associated with an approximately twofold increased risk of dementia [1,2]. This association could be explained by complex mechanisms, including systemic insulin resistance, advanced glycation end-products, microvascular endothelial dysfunction, and neuroinflammation [3]. Meanwhile, the polygenic risk score (PRS) quantifies an individual’s genetic predisposition to specific traits or diseases, offering potential for precision prevention [4]. This raises critical questions regarding targeting individuals genetically predisposed to type 2 diabetes for dementia prevention. Evidence indeed has demonstrated a shared genetic variability between type 2 diabetes and dementia, specifically Alzheimer’s disease (AD) [5], and a linear association between PRS for type 2 diabetes and an increased risk of vascular dementia (VaD) [6]. Critical inquiries remain on whether there is a dose–response association between PRS for type 2 diabetes and dementia that would allow us to establish a genetic threshold for prevention. Furthermore, type 2 diabetes has been shown to increase the risk of dementia, particularly among *APOE* ε4 carriers and women [7,8], and its impact varies by age at diabetes onset [9]. *APOE* ε4 status, sex, and age at diabetes onset should therefore be considered in dementia-related research [9,10].

Because the predictive factors for a disease extend beyond genetic factors, the application of PRS for type 2 diabetes in dementia prevention requires careful evaluation and thorough investigation [11,12]. Despite strong evidence supporting multidomain lifestyle interventions for dementia [13], tailored approaches for genetically at-risk individuals remain limited, highlighting a critical gap in efforts to mitigate genetic risk through lifestyle modification. A recent systematic review has shown that healthy lifestyle recommendations, developed by the American Heart Association (AHA), are associated with slower cognitive decline and lower risk of dementia [14]. Life’s Essential 8 (LE8), an updated AHA recommendation, includes eight health and lifestyle factors, i.e., managing weight, cholesterol, blood pressure, and blood glucose, alongside engaging in regular physical activity, maintaining a healthy diet, avoiding smoking, and obtaining adequate sleep [15]. A study using UK Biobank data found that maintaining a high LE8 score was associated with a lower risk of dementia, even among individuals carrying the *APOE* ε4 allele [16], suggesting that LE8 may mitigate the genetic risk of dementia. However, this relationship, particularly in the context of PRS for type 2 diabetes and dementia, has been rarely studied. Although a cohort study has reported a joint association between LE8 and genetic risk in relation to dementia incidence [17], further research is needed to clarify the role of LE8 in the association between type 2 diabetes PRS and dementia. This line of inquiry may also support the development of integrated prevention strategies targeting both dementia and diabetes.

In summary, important knowledge gaps remain in the current literature. Specifically, nonlinear dose–response relationships between type 2 diabetes PRS and dementia have not been evaluated, empirical threshold analyses to define genetic cut-offs for targeted dementia prevention are lacking, and evidence is scarce regarding whether comprehensive LE8 metrics interact protectively with a high type 2 diabetes PRS. Based on these knowledge gaps, we hypothesized that: (1) there is a nonlinear relationship between the type 2 diabetes PRS and incident dementia, with risks rising increasing evidently at extreme levels of polygenic loads, and this association may vary by sex, *APOE* ε4 status, and age of at diabetes onset; and (2) adherence to favorable LE8 metrics may be related to a lower dementia risk associated among individuals with high genetic susceptibility. To address these hypotheses, we analyzed data from using a cohort of 437,732 UK Biobank participants aged 40 years and older and above and followed, with an average follow-up period of 13 years, This study aimed to examine the association between PRS for type 2 diabetes and incident dementia, with particular attention to dose–response patterns, dementia subtypes, sex, *APOE* ε4 allele status, and age at diabetes onset. We also aimed to assess the preventive potential of LE8 by evaluating how LE8 modifies the genetic risk of dementia and by estimating the population-attributable fraction (PAF) if high, sustained LE8 scores were maintained.

## 2. Materials and Methods

### 2.1. Study Design and Participants

This prospective longitudinal study is based on data from the UK Biobank cohort (https://www.ukbiobank.ac.uk/ (accessed on 15 May 2026)) [18]. In brief, over 500,000 participants across England, Scotland, and Wales took part in the baseline examination in the UK Biobank between 2006 and 2010 [19]. In our study, follow-up information was obtained through registry linkages until March 2023. Among the baseline participants, we excluded individuals who withdrew or lacked follow-up data (*n* = 49,686), had a dementia diagnosis before or at baseline (*n* = 258), or were missing type 2 diabetes PRS data (*n* = 14,706), resulting in a final sample of 437,732 individuals (Figure S1).

### 2.2. Polygenic Risk Score for Type 2 Diabetes

The UK Biobank consists of a standard PRS for 28 diseases including type 2 diabetes, their genetic variants and corresponding weight scores, trained using external genome-wide association studies [20,21]. In the UK Biobank, genetic variants used for calculating the PRS were filtered based on quality metrics (e.g., minor allele frequency > 0.0005, imputation quality score > 0.1), and adherence to Hardy–Weinberg Equilibrium. The PRS for type 2 diabetes was calculated using trait-specific meta-analyses with a general Bayesian approach, combining data across multiple ancestries where appropriate and related to type 2 diabetes. The raw PRS was further normalized using the principal component-based ancestry centering step [21]. Considering that individuals in the top 5% of PRS exhibited significantly increased relevant disease risk and mortality [11,22], we defined high PRS for type 2 diabetes as scores at or above the 95th percentile, and low PRS as scores below the 95th percentile [22].

### 2.3. Life’s Essential 8

This study used a modified version of the original LE8 score, owing to the availability of diet data from the UK Biobank study [23]. The LE8 score was assessed based on behavioral and health components [15]. Each metric in the two components was assigned a score ranging from 0 to 100, with scores for non-high-density lipoprotein cholesterol and blood pressure further being subtracted by 20 points for drug treatment. Higher scores indicate a healthier status [15]. Detailed rules for score assignment were provided in Table S1. When calculating the average scores for total LE8, behavioral component, and health component, we used proration [23]. Specifically, participants with more than half of the metrics missing in a component were considered to have a missing score, while those with fewer than half of the metrics missing had their scores prorated. Specifically, if an individual had missing data for a single metric but complete data for more than 50% of the metrics within that health or behavioral component, their total score was calculated as the mean of the available metrics. The same methods were applied to calculate the metric score for diet patterns (Table S2). We categorized LE8 scores into low (LE8 score < 50) and moderate-to-high (≥50) levels [15]. The same thresholds were applied for behavioral and health components.

### 2.4. Diagnosis of Dementia

Follow-up data on dementia diagnosis were obtained through linkage to health and death records up until March 2023. All-cause dementia and subtypes of dementia were obtained according to algorithmically defined cases (Fields ID 42018–42025), including the first-occurrence data reporting dementia onsets within mental and behavioral disorders (Fields ID 130836–130843), hospital inpatient record (Fields ID 41270-71, 41280-81), death register (Fields ID 40001-02), and primary care data recorded (Field 42040) [19]. The current study included the onset of all-cause dementia, AD, VaD, and frontotemporal dementia (FTD). The codes of the International Classification of Diseases are provided in Table S3.

### 2.5. Demographic Factors, Health Conditions, and APOE ε4 Genotype

The age of participants at baseline was calculated using their birth date and the assessment date recorded at the examination center. Biological sex was categorized as women versus men. Educational attainment was classified into five groups based on the highest level of educational qualification reported: (1) no qualification, (2) Ordinary Levels, General Certificate of Secondary Education, General Certificate of Education, or equivalent, (3) Advanced level or equivalent, (4) other professional qualifications such as nursing and teaching, (5) college/university degree, National Vocational Qualification, Higher National Diploma/Certificates or equivalent [24]. Ethnicity was categorized into white and non-white [25]. Social deprivation was measured using the composite Townsend Deprivation Index, which considers unemployment, overcrowded household, non-car ownership, and non-home ownership [25]. Standard alcohol consumption was measured as the average weekly intake of red wine, white wine, beer, spirits, and other alcoholic beverages. Heavy alcohol consumption was defined as >35 standardized units per week for women and >50 units per week for men [26]. The somatic health was estimated using the Charlson Comorbidity Index, which summarizes 19 medical conditions based on UK Biobank Inpatient data using ICD-10 codes [27]. Mental disorders were identified from patient registers and included depression, anxiety, stress-related disorders, and traumatic brain injury. Diabetes onset was classified into early-onset versus late-onset utilizing age 40 as the threshold, according to evidence from cohort studies and the UK National Diabetes Audit. (Figure S2) [28,29,30]. The genetic variants rs429358 and rs7412, which followed the Hardy–Weinberg equilibrium, were used to define the three main *APOE* alleles (ε4, ε3, and ε2). We further assigned our participants to *APOE* ε4 carrier and non-carrier.

### 2.6. Statistical Analysis

Baseline characteristics were compared by incident all-cause dementia status, PRS levels, and LE8 scores using *t*-tests for continuous variables and chi-square tests for categorical variables. To assess potential bias from missing data, we compared baseline characteristics between participants with (*n* = 406,351) and without (*n* = 49,686) follow-up information.

Follow-up time was calculated as the number of years from the baseline assessment to the earliest of the following: first incident dementia diagnosis, death, last assessment, or end of follow-up (March 2023) [19]. Cox proportional-hazard regression models were applied to investigate the association between PRS for type 2 diabetes and incident all-cause and cause-specific dementia risk. Effect modification by LE8 was assessed by including interaction terms (LE8/behavioral/health components × PRS for type 2 diabetes) in the models. A Bonferroni-corrected significance threshold of *p* < 0.0042 (0.05/12) was applied for multiple comparisons. Stratified analyses were also conducted by *APOE* ε4 status, sex, and age of diabetes onset (40 vs. 40+). In addition, the dose–response relationships between PRS and dementia outcomes were evaluated using restricted cubic splines with knots at the 5th, 10th, 90th, and 95th percentiles, stratified by LE8 level, *APOE* ε4 status, sex, and diabetes onset. Furthermore, the PAFs for dementia were estimated under scenarios where the general population, men, and women achieved moderate-to-high LE8 levels. PAFs were calculated as: PAF = Proportion of cases exposed × attributable proportion among exposed. The 95% confidence interval (CI) for PAF is calculated as: 95% CI = PAF ± 1.96 × $\sqrt{varience(PAF)}$, where $variance(PAF)={\left((HR-1)/HR)\right)}^{2}$ × proportion of cases exposed × (1 − proportion of cases exposed)/total number + (proportion of cases exposed × 1/${HR}^{2}$) × Standard deviation (log HR).

All the previously mentioned models were adjusted for age, sex, educational attainment, ethnicity, Townsend Deprivation Index, heavy alcohol drinking, Charlson Comorbidity Index, number of mental diseases, and *APOE* ε4 status. Additional analyses excluded participants who developed dementia or dropped out within the first three years to address reverse causality. The main analysis was then repeated, first restricting the sample to individuals of White ancestry to account for population differences in the PRS for type 2 diabetes, and then with random blood glucose removed from the LE8 assessment, in line with the AHA’s recommendation to use fasting glucose instead. Finally, the main analysis was repeated using the 24-h dietary recall score to assess diet within the LE8 framework. All analyses were performed using STATA (version 17) and RStudio (version 4.4.1).

## 3. Results

### 3.1. Baseline Characteristics

In the study sample of 437,732 participants, the average age at baseline was 57.38 years (standard deviation [SD] = 8.05 years), and 54.52% were women. The average follow-up period was 13.27 years (SD = 2.27 years). During the follow-up period, 9425 participants (2.15%) developed dementia, including 4117 AD, 2115 VaD, and 296 FTD. The mean age at all-cause dementia diagnosis was 64.69 years (SD = 4.75).

Individuals who developed dementia were older and more likely to be men, *APOE* ε4 carriers, White, socially deprived, less educated, and had more baseline somatic and mental health conditions compared to those who remained dementia-free (Table 1). They also had lower baseline scores in total LE8, as well as the behavioral and health components. No significant difference in PRS for type 2 diabetes was observed between dementia and non-dementia groups. Notably, those with high PRS for type 2 diabetes had more somatic and mental illnesses, lower health scores, but higher total and behavioral LE8 scores than those with low PRS (Table S4). High LE8 scores correlated with fewer comorbidities and lower PRS. Participants lost to follow-up were younger, predominantly to be men, and non-White, yet exhibited healthier clinical and genetic profiles than those who remained in the study (Tables S5 and S6).

### 3.2. Relationship Between Polygenic Risk Score for Type 2 Diabetes and Dementia

**Figure 1:**
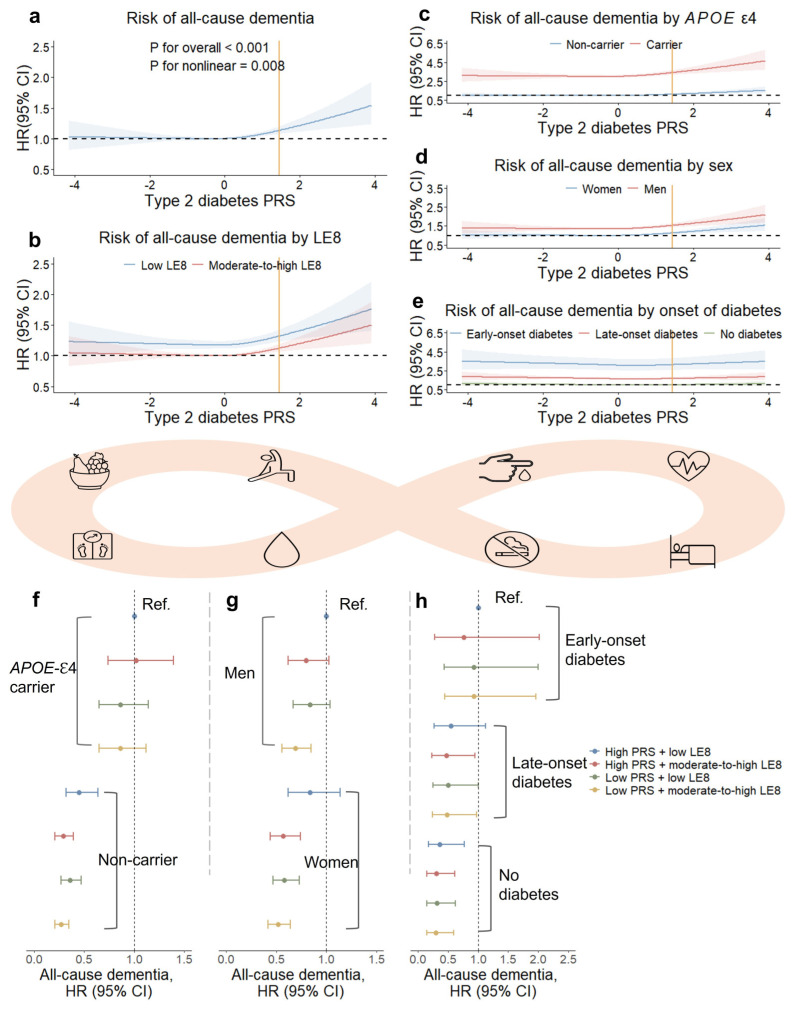
Association between PRS for type 2 diabetes and risk of all-cause dementia. Note: The vertical lines in panels (**a**–**e**) illustrate the 95% percentile of polygenic risk score for type 2 diabetes (1.45). The **upper** panels illustrate the non-linear relationship between PRS for type 2 diabetes and all-cause dementia risk in (**a**) the total study sample and stratified by (**b**) LE8 score, (**c**) *APOE* ε4 status, (**d**) sex, and (**e**) age at diabetes onset. The **lower** panels illustrate the joint association of PRS for type 2 diabetes and LE8 on dementia risk, stratified by (**f**) *APOE* ε4 status, (**g**) sex, and (**h**) age at diabetes onset. Abbreviations: HR: hazard ratio; CI: confidence interval; PRS: polygenic risk score; LE8: Life’s essential 8.

The Schoenfeld residuals test confirmed that the proportional hazard assumption was met for the PRS for type 2 diabetes across all outcomes, including all-cause dementia (ρ = −0.007, χ^2^ = 0.46, *p* = 0.50), AD (ρ = −0.026, χ^2^ = 2.87, *p* = 0.90), VaD (ρ = −0.000, χ^2^ = 0.00, *p* = 0.99), or FTD (ρ = −0.113, χ^2^ = 3.79, *p* = 0.05). This indicates that the association between PRS for type 2 diabetes and dementia incidence remained stable over the follow-up duration, justifying the use of a Cox model. Multivariable adjusted Cox models suggested that a higher PRS for type 2 diabetes was associated with an increased risk of all-cause dementia, AD, and VaD (HR and 95% CI: 1.04 [1.01–1.06], 1.04 [1.01–1.08], 1.08 [1.03–1.13]), but not with the risk of FTD (1.00 [0.89–1.12]) (Table S7, Figure S3). When restricted cubic splines were introduced, a dose–response relationship between PRS for type 2 diabetes and risk of all-cause dementia, especially VaD, was further observed (*p* for nonlinear < 0.05, Figure 1a and Figure S5a). Specifically, the HRs of all-cause and vascular dementia increased substantially when PRS reached approximately the 95th percentile and beyond (HR = 1.14, 95% CI = 1.08–1.20). Figures S4a and S6a showed the results for AD and FTD.

### 3.3. Relationship Between Polygenic Risk Score for Type 2 Diabetes and Dementia by LE8, APOE ε4, Sex, and Age at Diabetes Diagnosis

No significant interaction terms between PRS for type 2 diabetes and LE8 total, behavioral, or health scores on dementia risk were observed in the multivariable-adjusted Cox regression models after the Bonferroni correction (*p* > 0.0042). The restricted cubic splines showed that compared to individuals with a moderate-to-high LE8 score, those with a low LE8 score consistently exhibited a higher risk of all-cause dementia, especially VaD, across the entire range of PRS levels. Stratification analysis, dividing participants into low and moderate-to-high LE8 levels, revealed a similar dose–response relationship between PRS for type 2 diabetes and all-cause dementia and VaD, showing an exponential growth pattern in both groups (Figure 1b and Figure S5b). The HRs for all-cause dementia and VaD in relation to PRS for type 2 diabetes at its 95th percentile were 1.32 (95% CI: 1.23–1.42) and 1.96 (95% CI: 1.70–2.27) among individuals with a low LE8 score, whereas it decreased to 1.13 (95% CI: 1.06–1.19) and 1.23 (95% CI: 1.00–1.38) for those with a moderate-to-high LE8 score (Figure 1b).

Stratification analyses by *APOE* ε4 status suggested that *APOE* ε4 non-carriers exhibited a lower risk of all-cause dementia, AD, and VaD across the entire range of type 2 diabetes PRS levels, compared to carriers (Figure 1c, Figures S4c and S5c). This phenomenon was not observed for the outcome of FTD (Figure S6c). Among ε4 non-carriers, the risk of all-cause dementia and VaD remained low up to the 95th percentile of PRS for type 2 diabetes, while AD risk stayed low even beyond that threshold. (Figure 1c, Figures S4c and S5c). When type 2 diabetes PRS and LE8 were jointly considered, with *APOE* ε4 carriers with high PRS and low LE8 serving as the reference group, all-cause dementia risk was significantly lower among non-carriers. Among non-carriers, no substantial differences were observed across PRS or LE8 strata (Figure 1f). Similar patterns were observed for AD (Figure S4f).

Men exhibited a higher risk of all-cause dementia and VaD than women across the entire range of the nonlinear association between type 2 diabetes PRS and dementia risk (Figure 1d and Figure S5c). Further analysis combining PRS and LE8 levels revealed that, compared to men with high PRS and low LE8, women with high PRS and moderate-to-high LE8, or low PRS regardless of LE8, had significantly lower risks of all-cause dementia and AD (Figure 1g and Figure S4g). Women also exhibited lower VaD risk across all PRS and LE8 combinations relative to the reference group (Figure S5f).

Stratified analyses by diabetes onset showed that individuals with early-onset diabetes had consistently higher risks of all-cause dementia, AD, and VaD across all levels of PRS for type 2 diabetes PRS compared to those with late-onset diabetes. In contrast, individuals without diabetes had minimal risk for these outcomes, even beyond the 95th percentile of PRS (Figure 1e, Figures S4e and S5e). No significant difference in all-cause dementia risk was observed between early- and late-onset diabetes groups. Notably, compared to those with early-onset diabetes, high PRS, and low LE8, all non-diabetic subgroups showed significantly lower all-cause dementia risk across all PRS and LE8 combinations (Figure 1h). Similarly, all subgroups with late-onset or no diabetes showed lower VaD risk across all combinations (Figure S5h).

Very similar dose–response patterns were observed when participants were stratified based on their LE8 behavioral component scores (Figure S7). However, these patterns were not observed when stratification was based on the LE8 health component score (Figure S8). There was no significant difference in HRs for AD or FTD at the 95% percentile of the PRS between people with low or moderate-to-high LE8 (Figures S4 and S6).

### 3.4. Population-Attributable Fraction for All-Cause Dementia Risk Reduction

**Figure 2:**
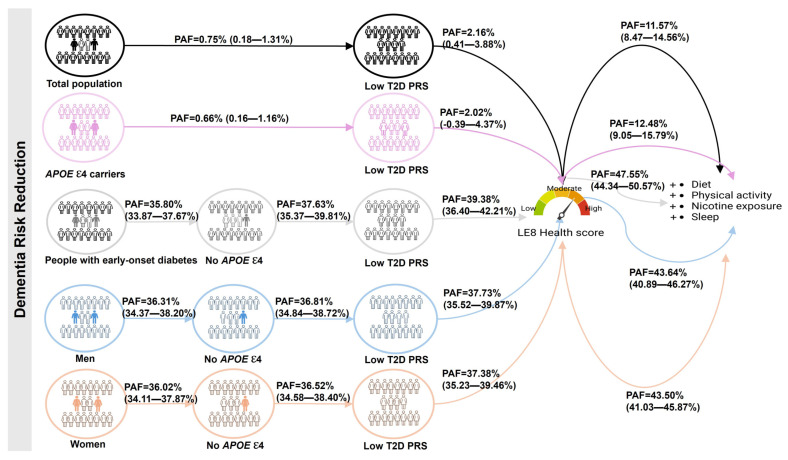
Population-attributable fraction for all-cause dementia risk reduction. Abbreviations: PRS: polygenic risk score; LE8: Life’s essential 8; PAF: population-attributable fraction.

In the total population, the PAF for all-cause dementia was 0.75% (95% CI: 0.18–1.31%) in the low PRS for type 2 diabetes scenario. This increased to 2.16% (0.41–3.88%) in the scenario additionally considering a moderate-to-high LE8 health score, and to 11.57% (8.47–14.56%) when all behavioral components were also at moderate-to-high levels (Figure 2). In the scenario of all men without the *APOE* ε4 allele, the population PAF was 36.31% (34.37–38.20%) and became 36.81% (34.84–38.72%) under low PRS. It increased to 43.64% (40.89–46.27%) when both LE8 health and behavioral components were at moderate-to-high levels (Figure 2). In the scenario of women without the *APOE* ε4 allele, with low PRS and moderate-to-high LE8 metrics, the PAF reached 43.50% (41.03–45.87%) (Figure 2).

### 3.5. Additional Analyses

The main findings remained unchanged after excluding individuals who developed dementia, died, or dropped out within the first three years (Figures S9–S12), after excluding non-White participants (*n* = 412,846, 94.31%; Figures S13–S16), when random blood glucose was excluded from the LE8 assessment (Figure S17), or when including the 24-h dietary recall data in the LE8 assessment (Figure S18).

## 4. Discussion

The findings from the current study can be summarized as follows. (1) A dose–response relationship exists between PRS for type 2 diabetes and risks of all-cause dementia and VaD, with risk sharply increasing beyond the 95th percentile. (2) *APOE* ε4 carriers, men, and those with early-onset diabetes consistently show a higher dementia risk than non-carriers, women, and those with late-onset or no diabetes. (3) People maintaining moderate-to-high LE8 scores, especially behavioral components, showed a reduced risk of all-cause dementia and VaD through all levels of PRS for type 2 diabetes than those with low scores. (4) Approximately 12% of all-cause dementia risk could be prevented in the scenario of individuals with low PRS for type 2 diabetes and moderate-to-high LE8 levels.

The population-based Malmö Diet and Cancer Study has identified a higher PRS for type 2 diabetes as a risk factor for both all-cause dementia and VaD [6], which is in line with our findings. In contrast to AD and VaD, the type 2 diabetes PRS was not associated with FTD. Unlike AD and VaD, which are associated with metabolic syndrome, insulin resistance, and microvascular pathology, FTD is predominantly tauopathy or RNA-binding proteinopathy with a distinct genetic architecture that separates from systemic metabolic dysfunction [31]. To our knowledge, this is the first study to identify the dose–response association, suggesting that a threshold effect of PRS on dementia risk may exist, similar to findings in cardiovascular diseases and dementia, where disease risk rises sharply beyond the top 20–25th percentiles of polygenic load [32,33]. These findings suggest that dementia risk does not increase steadily with rising polygenic load but instead follows a threshold model, where risk increases significantly once a critical genetic burden is reached. Screening asymptomatic individuals with polygenic risk scores in the highest 5% may help healthcare systems identify a subgroup at substantially elevated risk and prioritize them for early management of modifiable factors, including prediabetes. From a precision preventive medicine perspective, this approach could support earlier and more intensive lifestyle or clinical interventions for individuals whose genetic risk exceeds a clinically meaningful threshold.

Maintaining favorable LE8 behavioral components is associated with a lower dementia risk and slower brain aging, even in individuals with high genetic susceptibility, including those with the *APOE* ε4 or high PRS for dementia [34,35,36]. Our study builds on this by showing that individuals with a moderate-to-high behavioral score have a lower risk of dementia associated with PRS for type 2 diabetes compared to those with a low score. It is critical to note that formal multiplicative interaction terms between the type 2 diabetes PRS and LE8 scores did not achieve statistical significance after Bonferroni correction (*p* > 0.0042). Therefore, our data do not support the claim that lifestyle factors modify the PRS’s relative hazard ratio. Instead, our stratified restricted cubic splines capture an important public health reality: the baseline hazard of dementia is evidently elevated in the low LE8 subpopulation. Favorable LE8 metrics should be interpreted as an absolute risk buffer rather than a relative statistical modifier of genetic susceptibility. The stratifying association with *APOE* ε4 supports previous findings that diabetic *APOE* ε4 carriers have a higher risk of dementia than non-diabetic ε4 carriers [37,38]. This suggests that *APOE* ε4 may be necessary for the genetic risk of type 2 diabetes to significantly impact dementia risk, with non-carriers largely protected. Thus, prevention efforts should prioritize *APOE* ε4 carriers with tailored strategies. Notably, our subgroup PAF estimates for men and women lacking the *APOE* e4 allele reached high ranges (36–44%). This magnitude warrants careful interpretation: within an *APOE* e4 non-carrier population, the dominant unmodifiable genetic driver of neurodegeneration is absent. The relative proportion of remaining dementia cases that are statistically driven by the cardiometabolic and behavioral factors captured by the LE8 metrics expands dramatically, explaining these high attributable fractions. The sex-stratified findings contrast with a previous pooled analysis on diabetes and dementia [8]. Among the UK Biobank participants, men appear to be more vulnerable to vascular damage than women [39], which may explain our results and highlight the need to focus prevention on men in the UK. Furthermore, type 2 diabetes PRS levels were comparable between individuals with early- and late-onset diabetes, suggesting that genetic risk alone may have a limited influence on dementia without the clinical manifestation of diabetes. The elevated dementia risk in early-onset diabetes is therefore unlikely to be driven solely by genetic factors, but rather by the prolonged exposure to hyperglycemia, vascular damage, and metabolic complications over time in early-onset diabetes [40]. Routine screening strategies that facilitate the early identification of type 2 diabetes or prediabetes in younger adults may enable timely glycemic management, potentially reducing cumulative metabolic burden and mitigating the risk of long-term neurocognitive decline.

Recent evidence suggests that higher PRS may accelerate disease progression by simultaneously affecting multiple biological pathways [41]. A high PRS for type 2 diabetes reflects the presence of genetic variants linked to vascular, inflammatory, and metabolic dysfunction [40]. Specifically, this genetic susceptibility is closely linked to stroke and cerebral small vessel diseases [36], potentially explaining the link between type 2 diabetes PRS and dementia, particularly VaD. Type 2 diabetes-related variants may also disrupt insulin signaling in the brain, contributing to insulin resistance, which is linked to impaired synaptic plasticity, memory, and neuronal survival, thereby promoting neurodegeneration [34]. Metabolic dysfunction associated with insulin resistance is closely related to cognitive impairment [42], creating a pathway between PRS for type 2 diabetes and dementia. Furthermore, healthy behaviors such as maintaining a healthy weight and a balanced diet can regulate metabolic function and insulin sensitivity [43]. This helps counterbalance the harmful effects associated with a high PRS for type 2 diabetes. In addition, lifestyle factors such as regular physical activity promote neuroplasticity and cognitive function by stimulating the release of growth factors and neurotransmitters [44]. This partly explains the reduced dementia risk of people maintaining moderate-to-high LE8 behavioral components. Collectively, these interconnected biological and clinical pathways highlight the potential value of multidomain prevention strategies that integrate vascular, metabolic, and lifestyle risk management to reduce dementia risk. The strengths of this study include its large sample size, long follow-up period, and integration of genetic, lifestyle, and clinical data, which enable comprehensive risk assessment. The use of restricted cubic splines enabled clear visualization of the dose–response relationship between PRS for type 2 diabetes and dementia.

Our study provides scientific and statistical evidence for a general non-linear association between the PRS for type 2 diabetes and dementia. We found no evidence that LE8 modifies the association between type 2 diabetes PRS and dementia risk. Although a substantial proportion of dementia risk remains attributable to the *APOE* ε4 allele, our findings suggest that approximately 5–10% of dementia cases at different population levels could potentially be prevented if individuals maintained healthy behaviors across key lifestyle domains, including diet, physical activity, sleep, and smoking. These findings provide important evidence to inform the design of population-level dementia prevention programs and the implementation of public health strategies aimed at reducing dementia burden. Recently, increasing attention has been given to the debate that statistically significant results may, in some cases, be misleading when interpreted in terms of their practical importance. This concern is particularly relevant to clinical significance, which focuses on the real-world implications of research findings and evaluates whether an observed difference or association is meaningful in clinical practice. Our findings are derived from a large observational study, and the reported CI suggests a reasonable degree of robustness and precision in the estimated associations. Nevertheless, caution is warranted when interpreting these results and translating them into medical decision-making.

Conversely, clinical relevance focuses on the practical implications of a finding in real-world contexts and determines whether an observed difference or relationship holds practical meaning. Recently, there have been issues and debates surrounding the idea of statistical significance as certain experts argue that its mathematical representation can be misleading when it comes to practical understanding. These experts propose the inclusion of additional measures like effect sizes and confidence intervals. A sound comprehension of both statistical and clinical dimensions is vital in order to ensure precise interpretation of data and facilitate well-informed decision-making in the practice of medicine. By doing so, it positively influences the health of individuals and communities.

Several limitations should be considered when interpreting the findings of this study. First, the observational design precludes causal inference regarding the relationship between polygenic risk for type 2 diabetes, LE8, and dementia risk. Although extensive adjustments and sensitivity analyses were performed, residual confounding from unmeasured behavioral, socioeconomic, metabolic, or environmental factors cannot be fully excluded. Second, the UK Biobank cohort is subject to healthy volunteer bias, as participants are generally healthier and less socioeconomically deprived than the broader population, potentially limiting external generalizability. Third, the use of a modified LE8 score due to data availability constraints, particularly regarding dietary assessment and blood glucose measurements. This may reduce comparability with studies applying the original AHA LE8 framework. In addition, our primary categorization of LE8 into low and moderate-to-high groups may introduce residual confounding. Individuals with low LE8 scores systematically exhibit higher baseline morbidities and distinct socio-demographic profiles. The potential for residual confounding from unmeasured socioeconomic and health factors cannot be eliminated. Fourth, dementia diagnoses were derived from registry-based records, which may have introduced diagnostic bias (e.g., misclassification) and under representative. This is particularly relevant to less common dementia subtypes such as FTD. Fifth, the PRS used in this study was primarily derived from discovery GWAS cohorts of European ancestry, which is a major systemic limitation. While we performed sensitivity analyses restricting the sample to individuals of verified European descent to minimize population stratification, this dependency significantly reduces the generalizability to ethnically and ancestrally diverse populations. Furthermore, although statistically significant, the observed hazard ratios were relatively modest, suggesting that the clinical impact of PRS at the individual level should be interpreted cautiously. Sixth, our PAF calculations must be interpreted with strict caution, as they fundamentally rely on counterfactual epidemiological assumptions that presume an association is causal, unaffected by other covariates, and realistically intervened. Finally, longitudinal changes in lifestyle behaviors, glycemic control, medication use, and cardiometabolic status during follow-up were not comprehensively captured, limiting the ability to assess dynamic interactions between genetic susceptibility and behavioral modifications over time.

The findings of this study outline future perspectives across clinical, mechanistic, and translational domains. First, while our observational data suggest the importance of managing cardiovascular health, randomized clinical trials are needed to validate this preventive potential. Future intervention studies should investigate whether personalized and multidomain cardiovascular health interventions, stratified by polygenic risk for type 2 diabetes, can effectively reduce or delay dementia incidence in genetically vulnerable individuals. Second, future research must delve deeper into the biological mechanisms linking polygenic susceptibility to type 2 diabetes with neurodegeneration, insulin resistance, microvascular dysfunction, and metabolic dysregulation. Incorporating circulating biomarkers (such as plasma *p*-tau, amyloid-β, and neurofilament light chain) together with longitudinal glycemic measures alongside detailed cognitive trajectories will be essential to improving early risk prediction. Third, given that the current PRS is disproportionately derived from European-ancestry populations, expanding these analyses to ethnically diverse global cohorts is a vital priority. Finally, from a translational perspective, integrating LE8 assessment with polygenic risk profiling offers a promising avenue for precision medicine. This combined approach could facilitate the development of precision prevention strategies, enabling healthcare systems to identify high-risk individuals early in life who could benefit from targeted behavioral and cardiovascular interventions.

## 5. Conclusions

In conclusion, this large prospective cohort study demonstrated a dose–response association between polygenic risk for type 2 diabetes and dementia risk, particularly vascular dementia, with risk increasing more markedly at higher levels of genetic susceptibility. *APOE* ε4 carriers, men, and individuals with early-onset diabetes consistently exhibited greater vulnerability to dementia across different PRS levels. Maintaining good LE8 behaviors is related to a reduced risk of dementia associated with PRS for type 2 diabetes. Collectively, the study reinforces the relevance of multidomain prevention strategies integrating genetic, metabolic, vascular, and behavioral factors, while highlighting the potential role of precision prevention approaches for identifying high-risk populations that may benefit from early targeted interventions.

## Figures and Tables

**Table 1:** Characteristics of study participants at baseline by the incidence of dementia.

Characteristics at Baseline	Total Sample *n* = 437,732	Incident Dementia		*p*-Value
		No, *n* = 428,307 (97.85%)	Yes, *n* = 9425 (2.15%)	
**Age (years)**	57.38 (8.05)	57.22 (8.03)	64.69 (4.75)	<0.001
**Women, *n* (%)**	238,645 (54.52)	234,123 (54.66)	4522 (47.98)	<0.001
Education, *n* (%) *				<0.001
No qualification	78,066 (17.83)	74,830 (17.47)	3236 (34.33)	
Ordinary level/GCSE/GCE or equivalent	115,975 (26.49)	113,981 (26.61)	1994 (21.16)	
Advanced/AS level or equivalent	47,750 (10.91)	46,988 (10.97)	762 (8.08)	
Other professional qualifications	23,089 (5.27)	22,505 (5.25)	584 (6.20)	
College or university degree, NVQ, HND or HNC	167,112 (38.18)	164,495 (38.41)	2617 (27.77)	
Missing	5740 (1.31)	5508 (1.29)	232 (2.46)	
Ethnicity, *n* (%) *				<0.001
White	412,846 (94.31)	403,866 (94.29)	8980 (95.28)	
Non-white	23,838 (5.45)	23,432 (5.47)	406 (4.31)	
*APOE ε4* allele, *n* (%) *				<0.001
Non-carriers	264,066 (60.33)	260,388 (60.79)	3678 (39.02)	
Carriers	105,613 (24.13)	101,389 (23.67)	4224 (44.82)	
Missing	68,053 (15.55)	66,530 (15.53)	1523 (16.16)	
Onset of diabetes, *n* (%) *				<0.001
Early-onset	2515 (0.57)	2410 (0.56)	105 (1.11)	
Late-onset	43,794 (10.00)	41,766 (9.75)	2028 (21.52)	
No diabetes	390,454 (89.20)	383,219 (89.47)	7235 (76.76)	
**Townsend deprivation index**	−1.30 (3.09)	−1.30 (3.09)	−0.99 (3.28)	<0.001
**Heavy alcohol drinking, *n* (%)**				
No	299,036 (68.31)	293,196 (68.45)	5840 (61.96)	<0.001
Yes	1631 (0.37)	1611 (0.38)	20 (0.21)	
**Charlson Comorbidity Index**	0.27 (0.91)	0.26 (0.90)	0.49 (1.14)	<0.001
**Number of mental diseases, *n* (%)**	0.15 (0.44)	0.15 (0.44)	0.25 (0.57)	<0.001
**PRS for type 2 diabetes**	−0.13 (0.96)	−0.13 (0.96)	−0.12 (0.97)	0.101
Life’s Essential 8 total score *	63.20 (12.82)	63.27 (12.82)	59.81 (12.42)	<0.001
Behavioral component score *	64.85 (16.51)	64.88 (16.50)	63.53 (17.25)	<0.001
Health component score *	61.59 (17.34)	61.70 (17.75)	56.24 (16.74)	<0.001

Abbreviation: PRS: polygenic risk score; GCSE: General Certificate of Secondary Education; GCE: General Certificate of Education; AS: advanced subsidiary; NVQ: National Vocational Qualification; HND: Higher National Diploma; HNC: Higher National Certificates. * Represents missing values in the variables. Missing values were 83,806 for education, 1048 for ethnicity, 68,053 for *APOE* ε4 allele, 768 for Life’s Essential 8 total score, 735 for behavioral component score, and 39 for health component score.

## Data Availability

UK Biobank data are available through a procedure described at https://www.ukbiobank.ac.uk/use-our-data/apply-for-access/ (accessed on 15 May 2026).

## Supplementary Materials

The following supporting information can be downloaded at: https://www.mdpi.com/article/10.3390/nu18132080/s1. Table S1. Definition and scoring approach for quantifying cardiovascular health. Table S2. Criterion of a healthy diet in the UK Biobank. Table S3. The 10th revision of International Classification of Diseases for cause-specific dementia. Table S4. Characteristics of study participants at baseline by the levels of PRS for type 2 diabetes. Table S5. Characteristics of study participants at baseline by the levels of Life’s Essential 8 total score. Table S6. Characteristics of study participants at baseline by the availability of follow-up data. Table S7. Association between polygenic risk score for type 2 diabetes and dementia risk. Figure S1. Flowchart of study participants in the UK Biobank. Figure S2. Distribution of polygenic risk score for type 2 diabetes by the onset of diabetes. Figure S3. Time-to-event probability of developing dementia across levels of polygenic risk score for type 2 diabetes. Figure S4. Association between polygenic risk score for type 2 diabetes and risk of Alzheimer’s disease. Figure S5. Association between polygenic risk score for type 2 diabetes and risk of vascular dementia. Figure S6. Association between polygenic risk score for type 2 diabetes and risk of frontotemporal dementia. Figure S7. Association between polygenic risk score for type 2 diabetes and cause-specific dementia by behavioral components. Figure S8. Association between polygenic risk score for type 2 diabetes and cause-specific dementia by health components. Figure S9. Association between polygenic risk score for type 2 diabetes and risk of all-cause dementia in the three-year landmark analysis. Figure S10. Association between polygenic risk score for type 2 diabetes and risk of Alzheimer’s disease in the three-year landmark analysis. Figure S11. Association between polygenic risk score for type 2 diabetes and risk of vascular dementia in the three-year landmark analysis. Figure S12. Association between polygenic risk score for type 2 diabetes and risk of frontotemporal dementia in the three-year landmark analysis. Figure S13. Association between polygenic risk score for type 2 diabetes and risk of all-cause dementia in people of White ethnic background. Figure S14. Association between polygenic risk score for type 2 diabetes and risk of Alzheimer’s disease in people of White ethnic background. Figure S15. Association between polygenic risk score for type 2 diabetes and risk of vascular dementia in people of White ethnic background. Figure S16. Association between polygenic risk score for type 2 diabetes and risk of frontotemporal dementia in people of White ethnic background. Figure S17. Association between polygenic risk score for type 2 diabetes and dementia by LE8 after excluding random blood glucose from LE8 assessment. Figure S18. Association between polygenic risk score for type 2 diabetes and dementia by LE8 when including 24-h dietary recall data in LE8 assessment.

## Abbreviations

The following abbreviations are used in this manuscript:

PRS	Polygenic risk score
LE8	Life’s Essential 8
AHA	American Heart Association
VaD	Vascular dementia
AD	Alzheimer’s disease
FTD	Frontotemporal dementia
PAF	Population-attributable fraction
HR	Hazard ratio
CI	Confidence interval
SD	Standardized deviation
