# Supplementary material for: Life’s Essential 8, Polygenic Risk for Type 2 Diabetes, and Dementia: Evidence from the UK Biobank
Source: Nutrients. 2026 Jun 25;18(13):2080. doi: 10.3390/nu18132080 (PMC13363298; doi:10.3390/nu18132080)
Supplement: Supplementary file 1 [file nutrients-18-02080-s001.zip › nutrients-4341196-supplementary.pdf]

Table S1. Definition and scoring approach for quantifying cardiovascular health

Metrics	UK Biobank Field	Scores	Definitions
**Behavioural factors**			
Diet pattern[a]	Category 100052, mentioned in the Table S2	100	Diet score ≥95th percentile
		80	75th – <95th percentiles
		50	50th – <75th percentiles
		25	25th – <50th percentiles
		0	1st – <25th percentiles
Physical activity	22038 and 22039	100	Metabolic equivalent task minutes per week for moderate/vigorous activity ≥150
		90	120 – <150
		80	90 – <120
		60	60 – <90
		40	30 – <60
		20	1 – <30
		0	0
Nicotine exposure[a]	20116, 22507, 2897, 6194, 20003, 1269, 34, 52	100	Never smoker
		75	Former smoker, quit > 5 years, or missing quitting years
		50	Former smoker, quit 1–5 years
		25	Former smoker, quit <1 year, or currently using nedocromil sodium
		0	Current smoker
		Subtract 20 points (unless the score is 0) for living with an active indoor smoker at home	
Sleep health *	1160	100	Sleep duration each 24 hours: 7- <9 hours
		90	9 – <10 hours
		70	6 – <7 hours
		40	5 – <6 or ≥10 hours
		20	4 – <5 hours
		0	<4 hours
**Health factors**			
Body mass index	21001	100	<25 kg/m2
		70	25 – <30 kg/m2
		30	30 – <35 kg/m2
		15	35 – <40 kg/m2
		0	≥40 kg/m2
Blood lipids	30690, 30760, 6177	100	Non-HDL-cholesterol <130 mg/dL
		60	130 – <160 mg/dL
		40	160 – <190 mg/dL
		20	190 – <220 mg/dL
		0	≥220 mg/dL
		Subtract 20 points for drug-treating level	
Blood glucose *	2443, 30740, 30750	100	No history of diabetes and blood glucose <100 mg/dL (or HbA1c < 39 mmol/mol)
		60	No diabetes and blood glucose 100 – 125 mg/dL (or HbA1c 39 – 46 mmol/mol)
		40	Diabetes with HbA1c < 53 mmol/mol
		30	Diabetes with HbA1c 53 – <64 mmol/mol
		20	Diabetes with HbA1c 64 – <75 mmol/mol
		10	Diabetes with HbA1c 75 – <86 mmol/mol
		0	Diabetes with HbA1c ≥86 mmol/mol
Blood pressure †	4080, 93, 4079, 94, 6177	100	SBP <120 mmHg, and DBP < 80 mmHg
		75	SBP 120 – <130 mmHg, and DBP < 80 mmHg
		50	SBP 130 – <140 mmHg, or DBP 80 – <90 mmHg
		25	SBP 140 – <160 mmHg, or DBP 90 – <100 mmHg
		0	SBP ≥160 mmHg, or DBP ≥ 100 mmHg
		Subtract 20 points for drug-treating level.	

Abbreviations: HDL: high-density lipoprotein; HbA1C: glycated hemoglobin; SBP: systolic blood pressure; DBP: diastolic blood pressure.

* Modified from the recommendation of the American Heart Association in 2022: Lloyd-Jones D M, et al. Circulation. 2022. DOI: 10.1161/CIR.0000000000001078.

Modified items: (1) When defining diet pattern, we used the diet points assessed from 12 food items, according to Beydoun's study, but not the Healthy Eating Index-2015 or the Index of Dietary Approaches to Stop Hypertension which was recommended by the American Heart Association. (2) When defining nicotine exposure, people who quit smoking while missing the information regarding quitting years (41,311 out of 437,732 people in the analytical sample) were assigned a score of 50. (3) When defining sleep health, our study used the total sleep duration of 24 hours, which is different from the average hours of sleep per night recommended by the American Heart Association. (4) When defining blood glucose, our study used the level of HbA1c with a unit of mmol/mol, but not %.

† We defined SBP as the average of two automatic SBP readings. If this average was missing, we prioritized the second reading to mitigate white coat syndrome (Pioli MR, et al. Integr Blood Press Control. 2018. DOI: 10.2147/IBPC.S152761.). If the second automatic reading was unavailable, we used the first automatic reading. If both automatic SBP readings were absent, we used the average of manually measured SBP values. If the average manual measurement was missing, we prioritized the second manual measurement; otherwise, we used the first. In the same way, we defined the DBP using the automated readings and manually measured values in order.

Table S2. Criterion of a healthy diet in the UK Biobank

Food item	UK Biobank Field	Definition of meeting criterion, or criterion to assign 1 point.
**Consume more**		
Fresh and dried fruits	1309 and 1319	Consumption of fresh and dried fruits ≥3 servings per day. One serving of fresh fruits = one piece. One serving of dried fruits = 3 pieces.
Salad/raw and cooked vegetables	1289 and 1299	Consumption of salad/raw and cooked vegetables ≥3 servings per day. One serving of cooked vegetables = 8 tablespoons. One serving of salad/raw vegetables = 12 tablespoons.
Whole grains		
Bread	1438 and 1448	Wholemeal or wholegrain bread ≥3 servings per day.
Cereal*	1458 and 1468	Daily bowls of whole wheat cereal (bran cereal, biscuit cereal, oat cereal, and muesli) ≥3 servings per day.
Oily and non-oily fish shellfish	1329 and 1339	Consumption of oily and non-oily fish shellfish ≥2 servings/week
Dairy products (cheese)*	1408	Reporting consumption of cheese once a day.
Vegetable oil	2654	Reporting use of olive oil or polyunsaturated/sunflower oil
**Consume less**		
Refined grains, starches, added sugars	1438 and 1448	Non-whole grains <1.5 servings per day
Processed meats	1349	Once a week or less.
Unprocessed red meats	1369, 1379, and 1389	Summation of frequency of consumption across three types of red meats (lamb/mutton, beef or pork) <3.
Industrial trans fat	1428	Never or rarely using spread.
Sugar-sweetened beverages	6144	Never eating sugar or food/drink containing sugar.
Sodium	1478	Never or rarely adding salad to food.

Modified from Beydoun H A, et al. Alzheimers Dement. 2023. DOI: 10.1002/alz.13405.

*Modified items: (1) Cereal was defined by variables 1458 and 1468 in our study, while 1458 and 1448 in Beydoun's study. (2) The dairy product met the criterion when participants reported the consumption of two milk items and eating cheese once a day, in Beydoun's study. However, the number of milk items was not available in UK Biobank. Therefore, we only used the cheese item to define the dairy product.

Table S3. The 10th revision of International Classification of Diseases for cause-specific dementia

Cause-specific dementia	ICD-10 Code	Description
**Alzheimer's Disease**	F00.0	Dementia in Alzheimer's disease with early onset
	F00.1	Dementia in Alzheimer's disease with late onset
	F00.2	Dementia in Alzheimer's disease, atypical or mixed type
	F00.9	Dementia in Alzheimer's disease, unspecified
	G30.0	Alzheimer's disease with early onset
	G30.1	Alzheimer's disease with late onset
	G30.8	Other Alzheimer's disease
	G30.9	Alzheimer's disease, unspecified
**Vascular Dementia**	F01.0	Vascular dementia of acute onset
	F01.1	Multi-infarct dementia
	F01.2	Subcortical vascular dementia
	F01.3	Mixed cortical and subcortical vascular dementia
	F01.8	Other vascular dementia
	F01.9	Vascular dementia, unspecified
	I67.3	Binswanger's disease
**Frontotemporal Dementia**	F02.0	Dementia in Picks disease
	G31.0	Circumscribed brain atrophy

Abbreviations: ICD-10: the 10th revision of International Classification of Diseases.

Table S4. Characteristics of study participants at baseline by the levels of PRS for type 2 diabetes

Characteristics at baseline	Total sample n= 437732	T2D PRS		*P*-value
		Low, n= 415845 (95.00%)	High, n=21887 (5.00%)	
**Age (years)**	57.38 (8.05)	57.41 (8.04)	56.94 (8.12)	<0.001
**Female, n (%)**	238645(54.52)	226718(54.52)	11927(54.49)	<0.001
**Education, n (%)**				<0.001
No qualification	78066 (17.83)	73673 (17.72)	4393 (20.07)	
Ordinary level/GCSE/CSE or equivalent	115975 (26.49)	110039 (26.46)	5936 (27.12)	
Advanced/AS level or equivalent	47750 (10.91)	45562 (10.96)	2188 (10.00)	
Other professional qualifications	23089 (5.27)	21935 (5.27)	1554 (5.27)	
College or university degree, NVQ, HND or HNC	167112 (38.18)	159249 (38.30)	7863 (35.93)	
**Ethnicity, n (%)**				<0.001
White	412846(94.31)	392987(94.50)	19859 (90.73)	
Non-white	23838 (5.45)	21876(5.26)	1962 (8.96)	
APOE-ε4 **allele, n (%)**				<0.001
Non-carriers	264066 (60.33)	250196 (60.17)	13870 (63.37)	
Carriers	105613 (24.13)	101038 (24.30)	4575 (20.90)	
**Townsend deprivation index**	-1.30 (3.09)	-1.31 (3.09)	-1.01 (3.23)	<0.001
**Heavy alcohol drinking, n (%)**				
No	299036 (68.31)	285399 (68.63)	13637 (62.31)	<0.001
Yes	1631 (0.37)	1581 (0.38)	50 (0.23)	
**Charlson Comorbidity Index**	0.27 (0.91)	0.26 (0.90)	0.37 (1.01)	<0.001
**Number of mental diseases, n (%)**	0.15 (0.44)	0.15 (0.44)	0.17 (0.46)	<0.001
**PRS for type 2 diabetes**	-0.13 (0.36)	-0.24 (0.86)	1.85 (0.36)	<0.001
**Life's Essential 8 total score**	63.20 (12.82)	63.27 (12.77)	59.82(13.18)	<0.001
**Behavioral component score**	47.85 (16.51)	64.90 (16.49)	64.08(16.94)	<0.001
**Health component score**	61.59 (17.34)	61.90 (17.66)	55.61(18.31)	<0.001

Abbreviation: PRS: polygenic risk score; GCSE: General Certificate of Secondary Education; GSE: General Certificate of Education; AS: advanced subsidiary; NVQ: National Vocational Qualification; HND: Higher National Diploma; HNC: Higher National Certificates.

Table S5. Characteristics of study participants at baseline by the levels of Life's Essential 8 total score

Characteristics at baseline	Total sample n= 436964	Life's Essential 8 total score		*P*-value
		Low, n= 65575 (15.01%)	Moderate-to-high, n=317389 (84.99%)	
**Age (years)**	57.38 (8.05)	58.19 (7.49)	57.17 (8.12)	<0.001
**Female, n (%)**	238247(54.52)	29087(44.36)	209160(56.32)	<0.001
**Education, n (%)**				<0.001
No qualification	77961 (17.84)	18999 (29.97)	58962 (15.88)	
Ordinary level/GCSE/CSE or equivalent	115950 (26.54)	17708 (27.00)	98242 (26.45)	
Advanced/AS level or equivalent	47744 (10.93)	6024 (9.19)	41720 (11.23)	
Other professional qualifications	23079 (10.93)	3496 (5.33)	19583 (5.27)	
College or university degree, NVQ, HND or HNC	167087 (38.24)	18307 (27.92)	148771 (40.06)	
**Ethnicity, n (%)**				<0.001
White	412706(94.45)	61729(94.13)	350977(94.50)	
Non-white	23668 (5.42)	3713 (5.66)	19955 (5.37)	
***APOE-ε4* allele, n (%)**				<0.001
Non-carriers	263541 (60.31)	37698 (57.49)	225843(60.81)	
Carriers	105423 (24.13)	15470 (23.59)	89953 (24.22)	
**Townsend deprivation index**	-1.30 (3.09)	-0.51 (3.38)	-1.44 (3.01)	<0.001
**Heavy alcohol drinking, n (%)**				
No	298977 (68.42)	41362 (63.08)	257615(69.37)	<0.001
Yes	1631 (0.37)	305 (0.47)	1326 (0.36)	
**Charlson Comorbidity Index**	0.27 (0.91)	0.24 (0.86)	0.44 (0.13)	<0.001
**Number of mental diseases, n (%)**	0.15 (0.44)	0.22 (0,53)	0.14 (0.42)	<0.001
**PRS for type 2 diabetes**	-0.13 (0.36)	0.07 (0.95)	-0.17 (0.96)	<0.001
**Life's Essential 8 total score**	63.20 (12.82)	42.65 (5.93)	66.82(9.97)	<0.001
**Behavioral component score**	64.85 (16.51)	44.28 (13.98)	68.49(14.09)	<0.001
**Health component score**	61.59 (17.74)	41.14 (12.98)	65.20(15.93)	<0.001

Abbreviation: PRS: polygenic risk score; GCSE: General Certificate of Secondary Education; GSE: General Certificate of Education; AS: advanced subsidiary; NVQ: National Vocational Qualification; HND: Higher National Diploma; HNC: Higher National Certificates.

Table S6. Characteristics of study participants at baseline by the availability of follow-up data

Characteristics at baseline	Total sample n= 502382	Follow-up		*P*-value
		Yes, n= 452696 (90.11%)	No, n=49686 (9.89%)	
**Age (years)**	57.03 (8.09)	57.37 (8.05)	53.91 (7.78)	<0.001
**Female, n (%)**	273311(54.40)	247589(54.69)	25722(51.77)	<0.001
**Education, n (%)**[a]				<0.001
No qualification	85258 (16.97)	80338 (17.75)	4920 (9.90)	
Ordinary level/GCSE/CSE or equivalent	132052 (26.29)	119018 (26.29)	13034 (26.23)	
Advanced/AS level or equivalent	55305 (11.01)	48936 (10.81)	6369 (12.82)	
Other professional qualifications	25799 (5.14)	23712 (5.24)	2087 (4.20)	
College or university degree, NVQ, HND or HNC	193837 (38.58)	171342 (37.85)	22495 (45.27)	
**Ethnicity, n (%)**				<0.001
White	472023(93.96)	426190 (94.14)	45833 (92.25)	
Non-white	28778 (5.73)	25090 (5.54)	3688 (7.42)	
APOE-ε4 **allele, n (%)**				<0.001
Non-carriers	295102 (58.74)	265266 (58.60)	29836 (60.05)	
Carriers	117797 (23.45)	106165 (23.45)	11632 (23.41)	
**Townsend deprivation index**	-1.29 (3.09)	-1.28 (3.10)	-1.42 (3.02)	<0.001
**Heavy alcohol drinking, n (%)**				
No	344319 (68.54)	308234 (68.09)	36085 (72.63)	<0.001
Yes	1897 (0.38)	1686 (0.37)	211 (0.42)	
**Charlson Comorbidity Index**	0.25 (0.88)	0.28 (0.92)	0.00 (0.02)	<0.001
**Number of mental diseases, n (%)**	0.14 (0.42)	0.15 (0.44)	0.00 (0.02)	<0.001
**PRS for type 2 diabetes**	-0.14 (0.96)	-0.13 (0.96)	-0.17 (0.96)	0.101
**Life's Essential 8 total score**	63.41 (12.84)	63.13 (12.87)	65.87(12.23)	<0.001
**Behavioral component score**	65.01 (16.46)	64.81 (16.55)	66.87(15.48)	<0.001
**Health component score**	61.79 (17.92)	61.45 (17.94)	64.91(17.41)	<0.001

Abbreviation: PRS: polygenic risk score; GCSE: General Certificate of Secondary Education; GSE: General Certificate of Education; AS: advanced subsidiary; NVQ: National Vocational Qualification; HND: Higher National Diploma; HNC: Higher National Certificates.

Table S7. Association between polygenic risk score for type 2 diabetes and dementia risk

**Polygenic risk score for type 2 diabetes**	**All-cause dementia**		**Alzheimer's disease**		**Vascular dementia**		**Frontotemporal dementia**	
	number §	HR (95%CI)	number	HR (95% CI)	number	HR (95%CI)	number	HR (95%CI)
**Continuous score**, n=485693								
	9425	1.04 (1.01, 1.06)‡	4177	1.04 (1.01, 1.08)†	2115	1.08 (1.03, 1.13)†	296	1.00 (0.89, 1.12)
**Categorical levels**								
Low, n=461409	8896	1 (ref)	3957	1 (ref)	1977	1 (ref)	285	1 (ref)
High, n=24284	529	1.20 (1.09, 1.30)‡	220	1.15 (1.01, 1.32)*	138	1.37 (1.15, 1.63)‡	11	0.78 (0.43, 1.42)

**P<0.05, †P<0.01, ‡P<0.001.*

Abbreviation: HR: hazards ratio; CI: confidence interval.

§ A total of 549 individuals were diagnosed with two types of dementia; among them, 496 had both AD and VaD, 53 had both AD and FTD, and 30 had both VaD and FTD. A total of 10 individuals had all three types of dementia examined in this study.

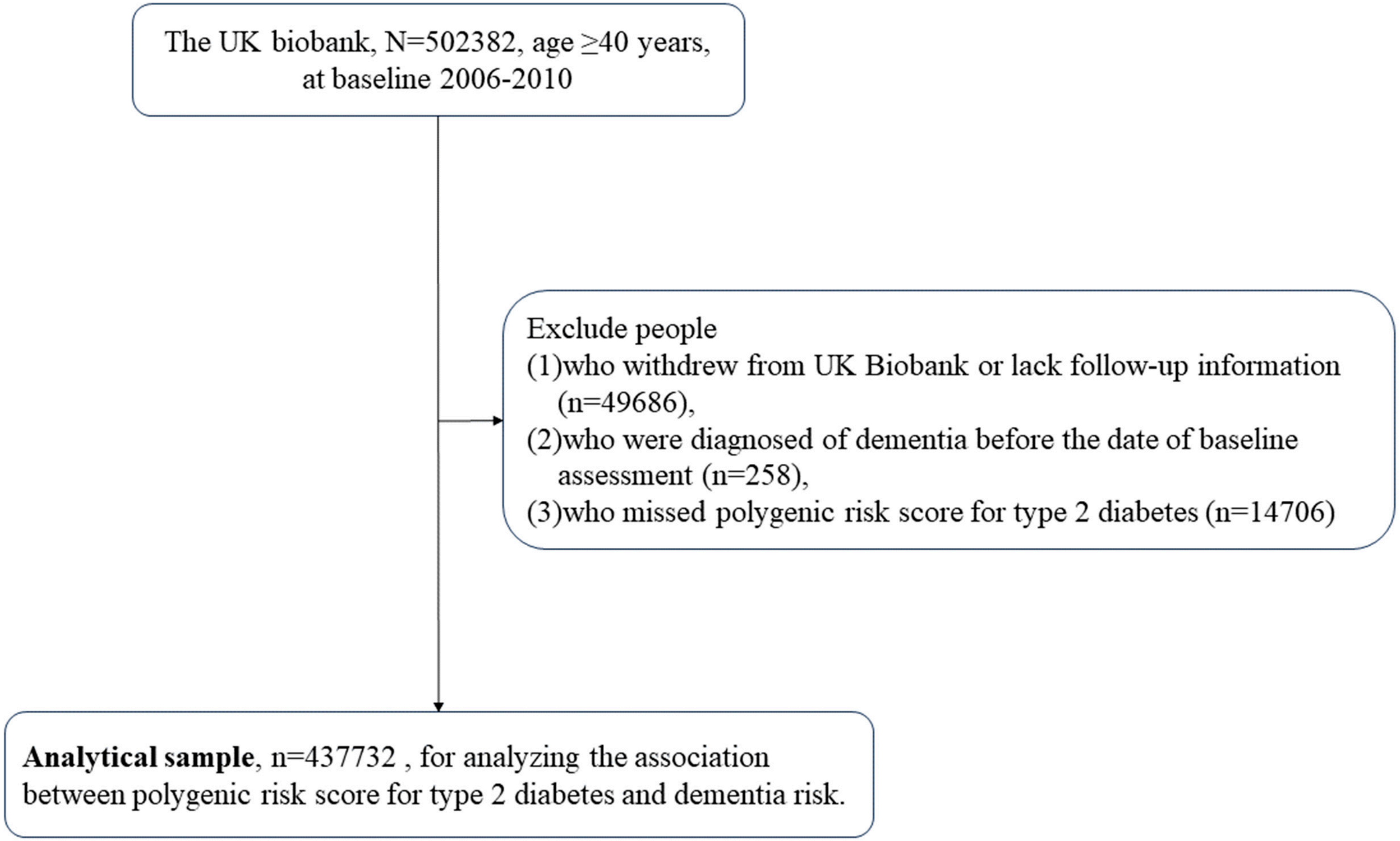

Figure S1. Flowchart of study participants in the UK Biobank

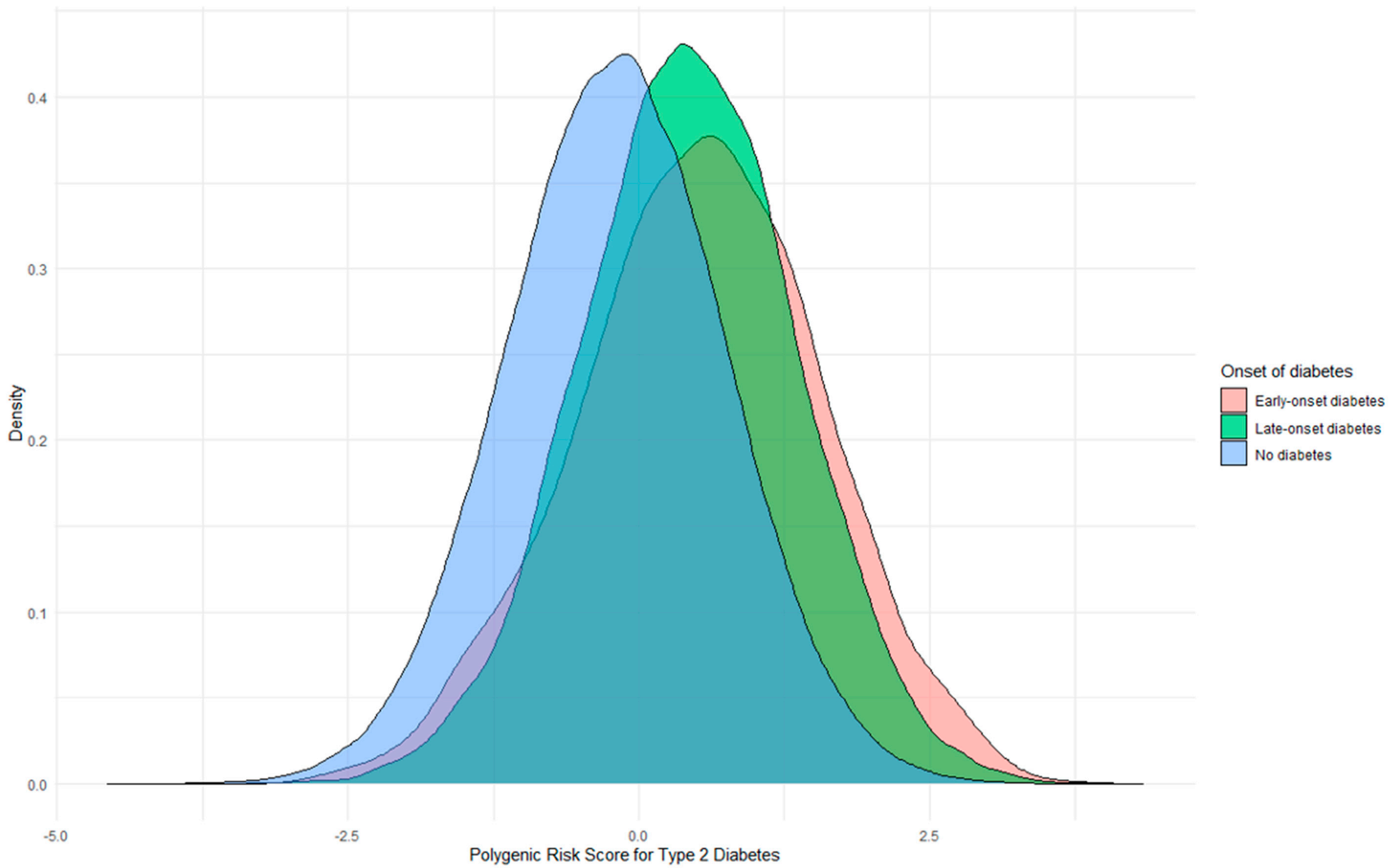

Figure S2. Distribution of polygenic risk score for type 2 diabetes by the onset of diabetes

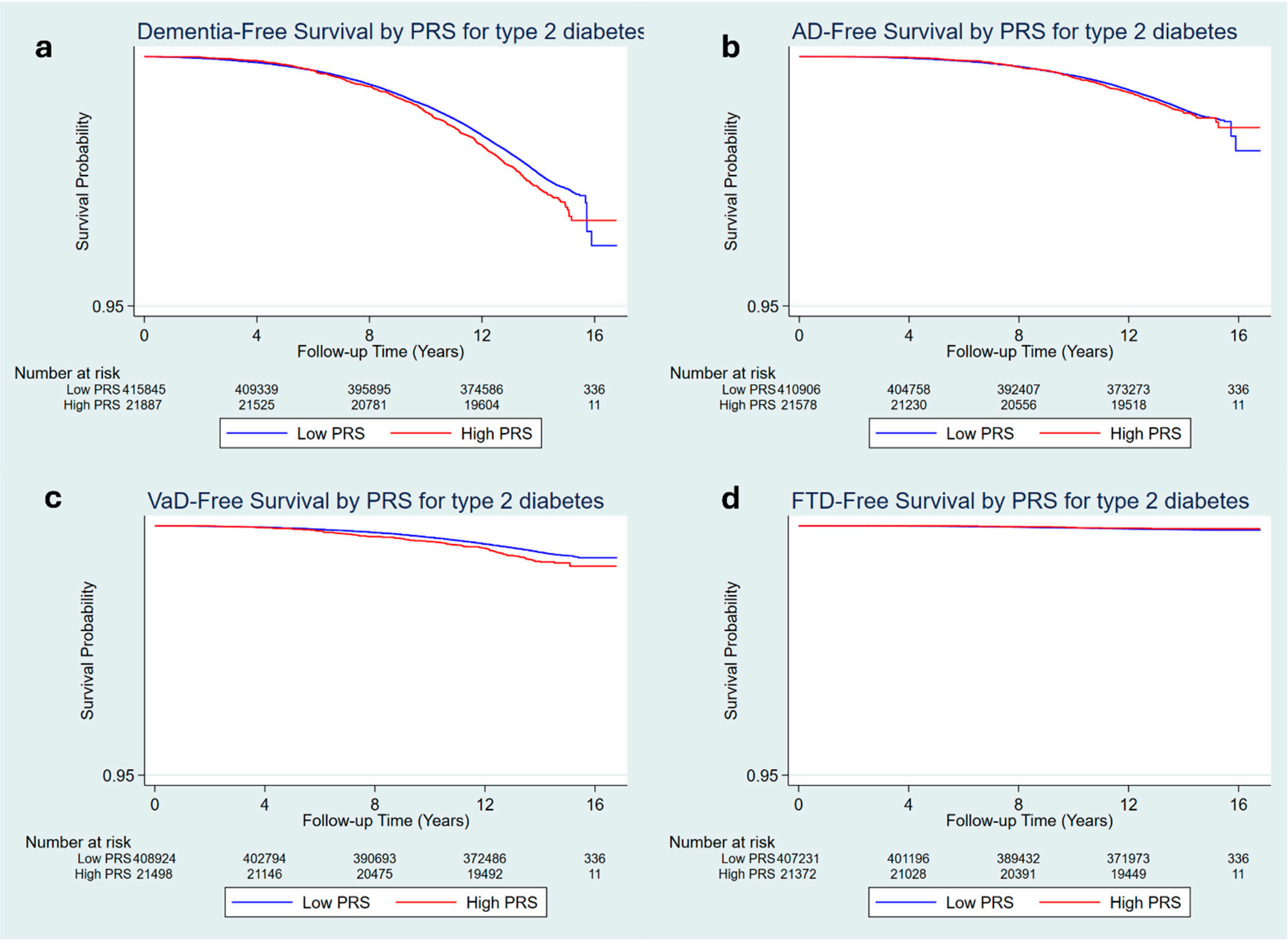

Figure S3. Time-to-event probability of developing dementia across levels of polygenic risk score for type 2 diabetes

Abbreviations**:** PRS: polygenic risk score. AD: Alzheimer's disease; VaD: vascular dementia; FTD: frontotemporal dementia.

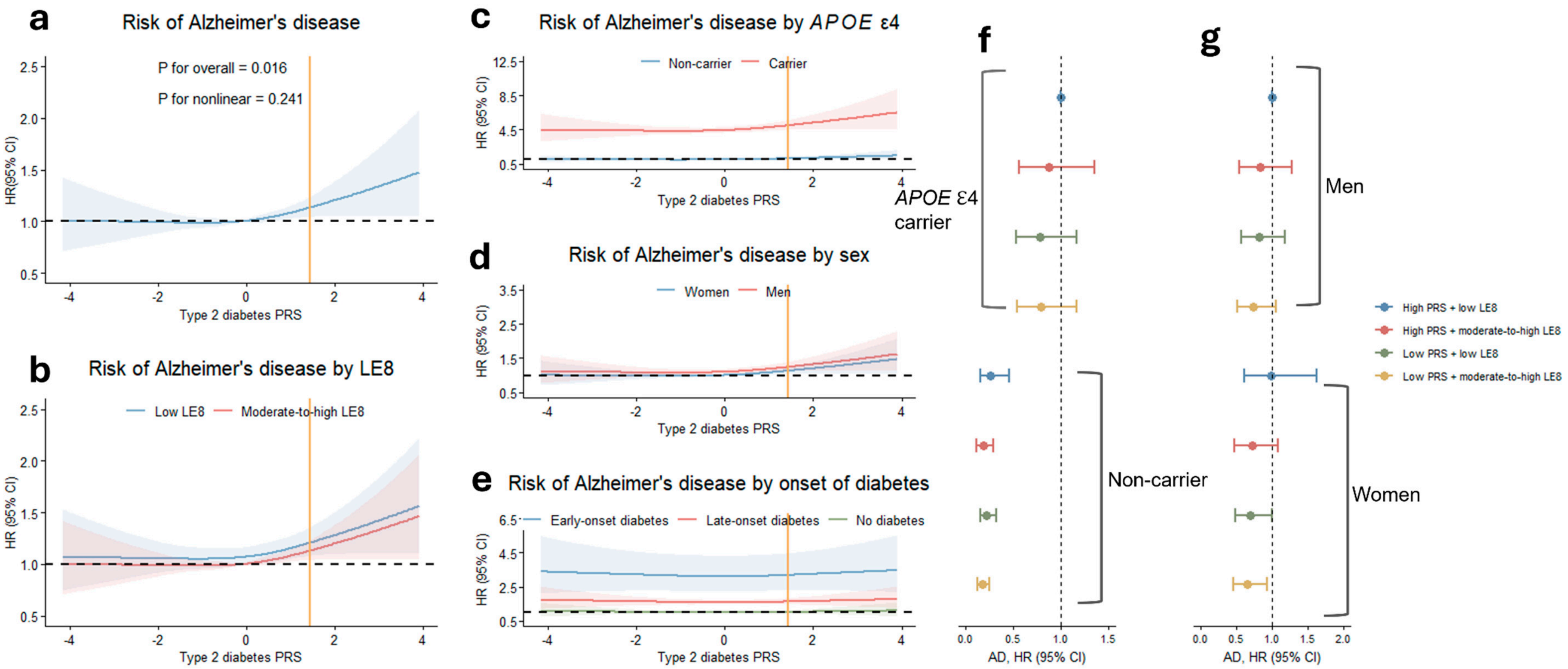

Figure S4. Association between polygenic risk score for type 2 diabetes and risk of Alzheimer's disease

Note: The pink vertical line in the graph indicates the 95% percentile of polygenic risk score for type 2 diabetes (1.45).

Abbreviations: HR: hazards ratio; CI: confidence interval; PRS: polygenic risk score; LE8: Life's essential 8; AD: Alzheimer's disease.

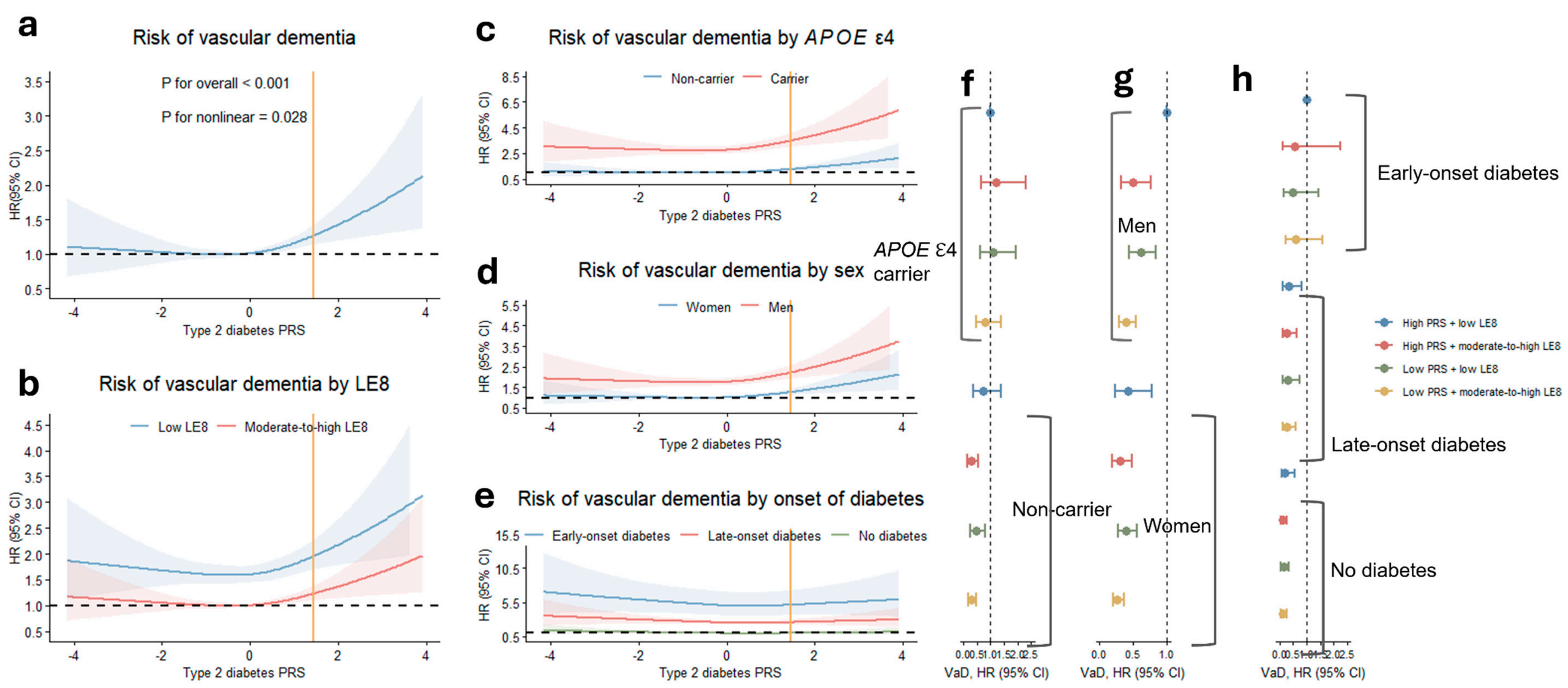

Figure S5. Association between polygenic risk score for type 2 diabetes and risk of vascular dementia

Note: The pink vertical line in the graph indicates the 95% percentile of polygenic risk score for type 2 diabetes (1.45).

Abbreviations: HR: hazards ratio; CI: confidence interval; T2D: type 2 diabetes; PRS: polygenic risk score; LE8: Life's essential 8; VaD: vascular dementia.

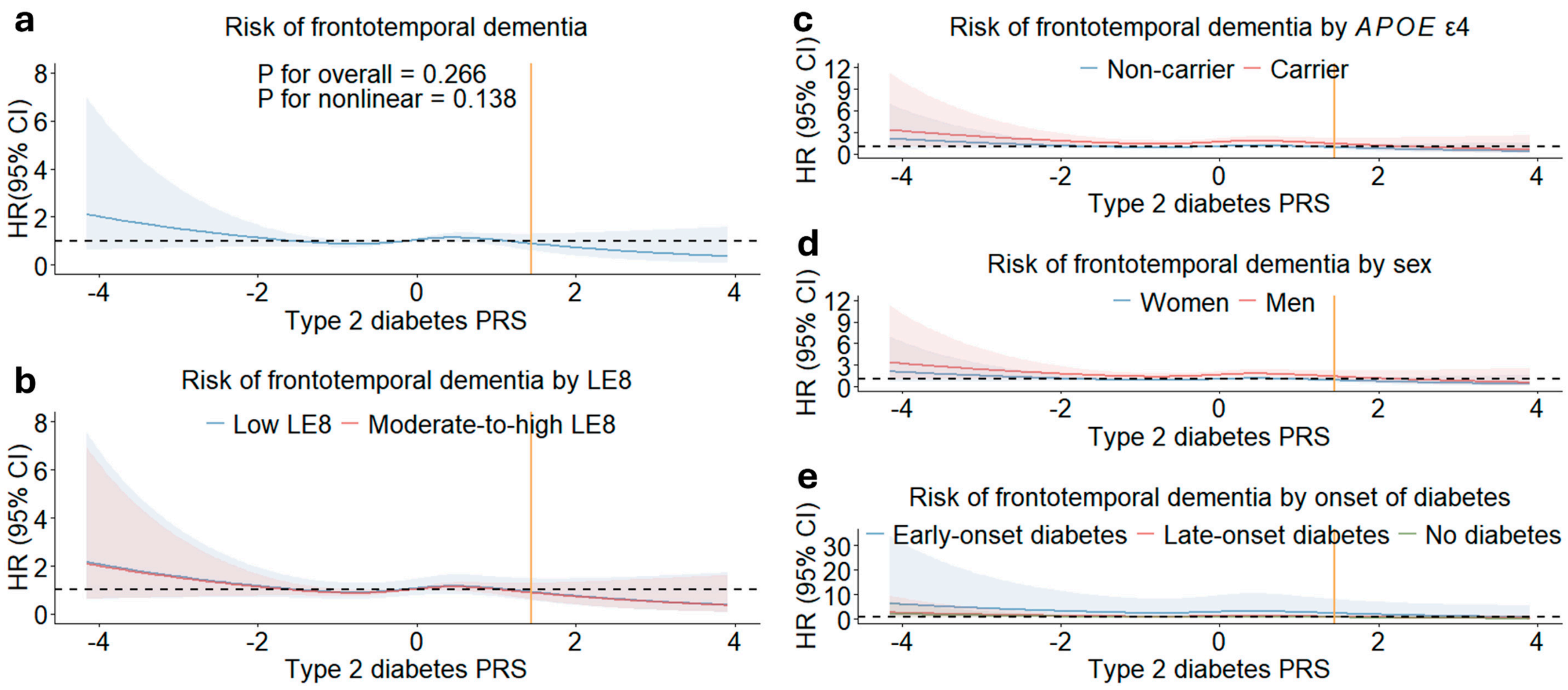

Figure S6. Association between polygenic risk score for type 2 diabetes and risk of frontotemporal dementia

Note: The pink vertical line in the graph indicates the 95% percentile of polygenic risk score for type 2 diabetes (1.45).

Abbreviations: HR: hazards ratio; CI: confidence interval; PRS: polygenic risk score; LE8: Life's essential 8.

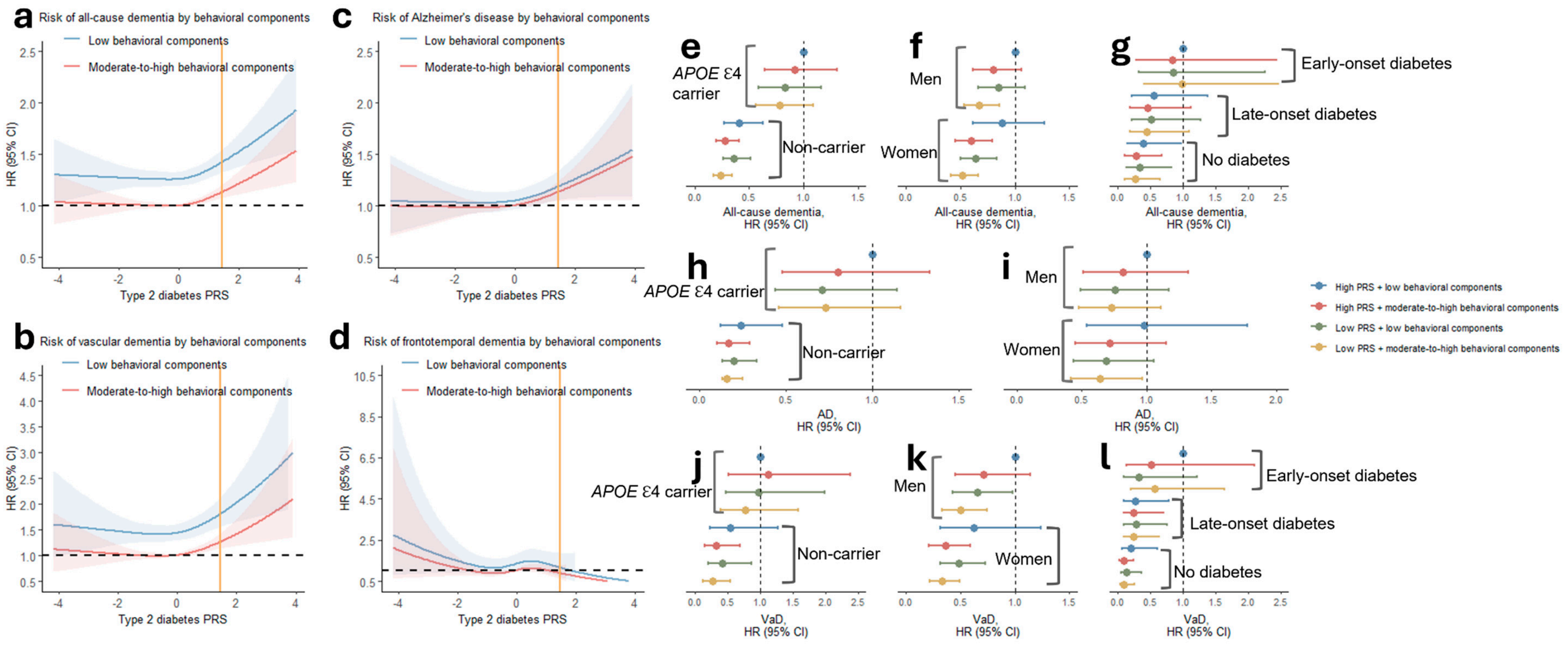

Figure S7. Association between polygenic risk score for type 2 diabetes and cause-specific dementia by behavioral components

Note: The pink vertical line in the graph indicates the 95% percentile of polygenic risk score for type 2 diabetes (1.45).

Abbreviations: HR: hazards ratio; CI: confidence interval; PRS: polygenic risk score; LE8: Life's essential 8; AD: Alzheimer's disease; VAD: vascular dementia.

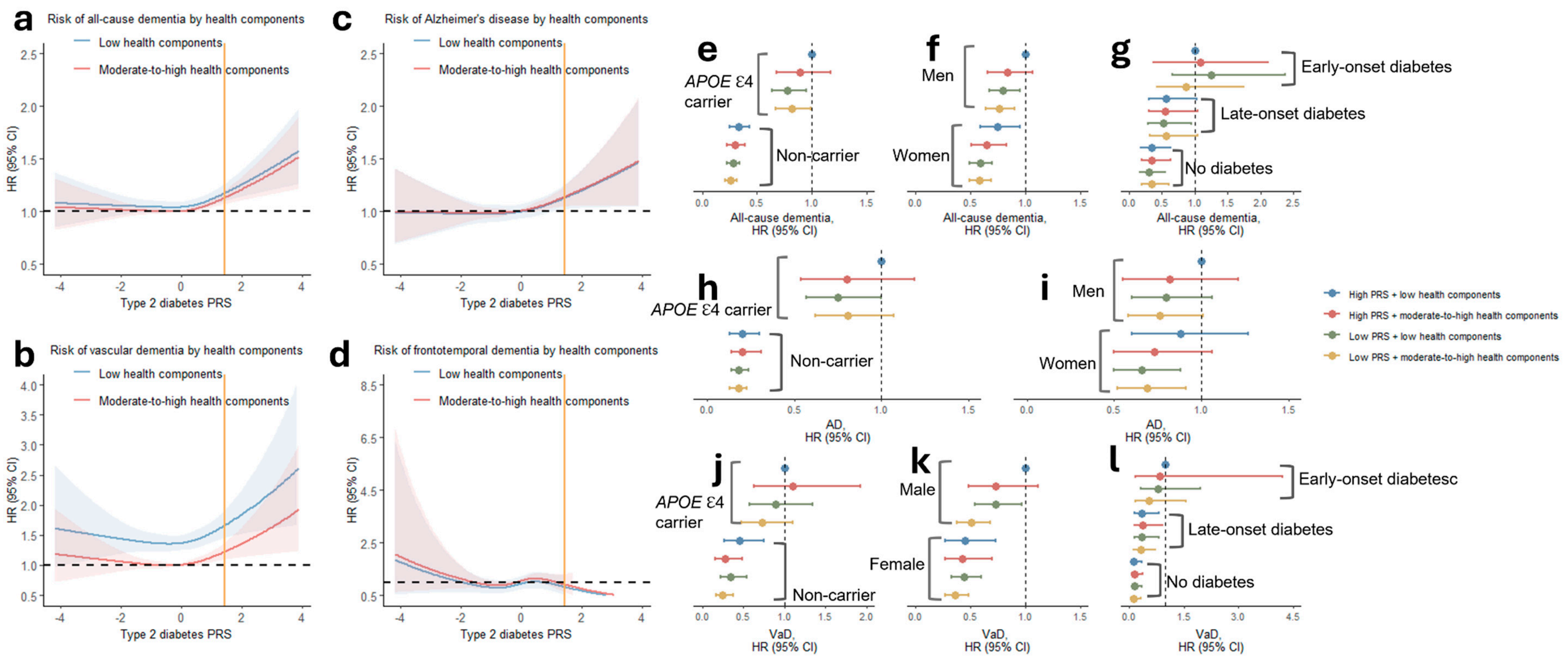

Figure S8. Association between polygenic risk score for type 2 diabetes and cause-specific dementia by health components

Note: The pink vertical line in the graph indicates the 95% percentile of polygenic risk score for type 2 diabetes (1.45).

Abbreviations: HR: hazards ratio; CI: confidence interval; PRS: polygenic risk score; LE8: Life's essential 8; AD: Alzheimer's disease; VAD: vascular dementia.

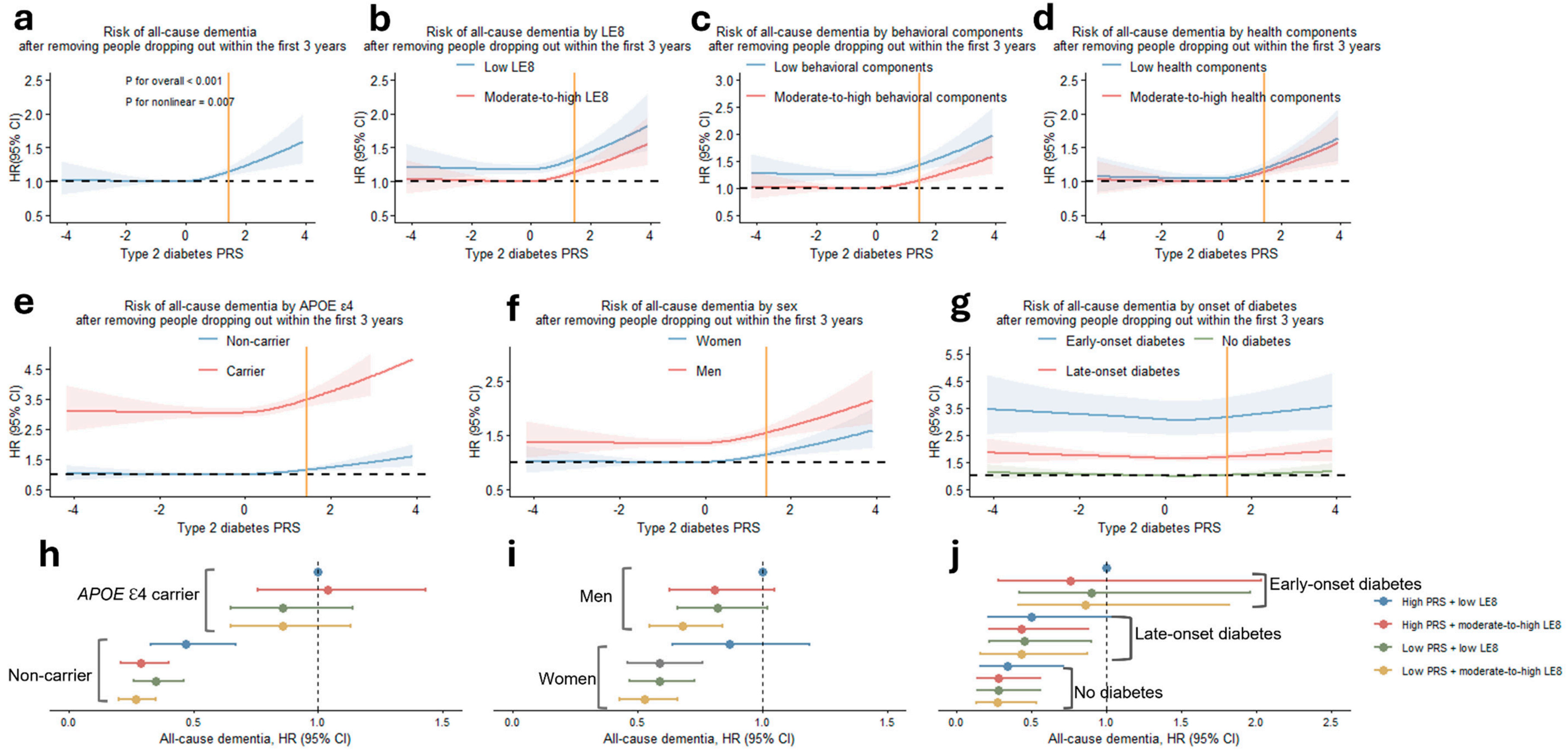

Figure S9. Association between polygenic risk score for type 2 diabetes and risk of all-cause dementia in the three-year landmark analysis

Note: The pink vertical line in the graph indicates the 95% percentile of polygenic risk score for type 2 diabetes (1.45).

Abbreviations: HR: hazards ratio; CI: confidence interval; PRS: polygenic risk score; LE8: Life's essential 8.

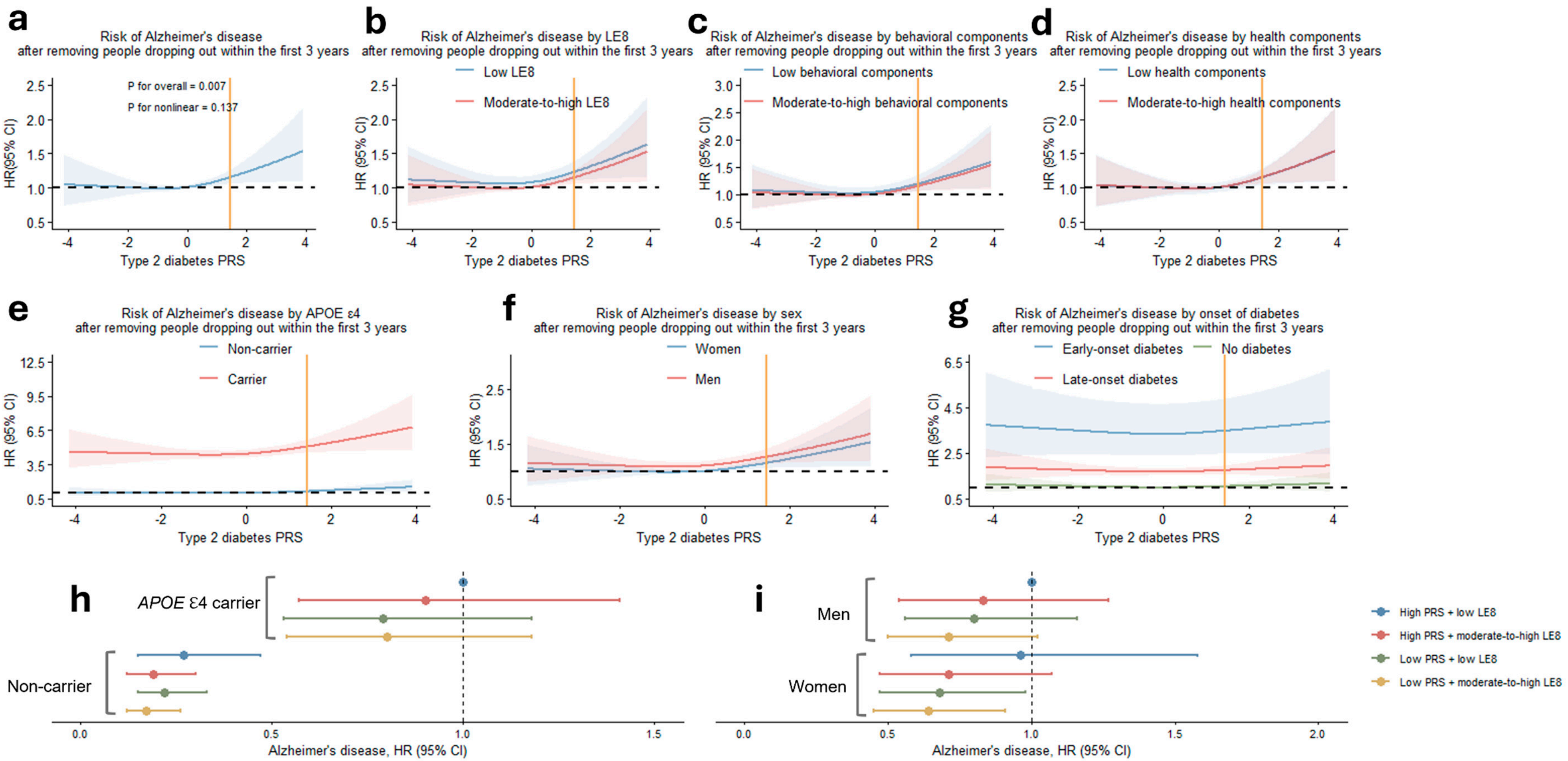

Figure S10. Association between polygenic risk score for type 2 diabetes and risk of Alzheimer's disease in the three-year landmark analysis

Note: The pink vertical line in the graph indicates the 95% percentile of polygenic risk score for type 2 diabetes (1.43).

Abbreviations: HR: hazards ratio; CI: confidence interval; PRS: polygenic risk score; AD: Alzheimer's disease.

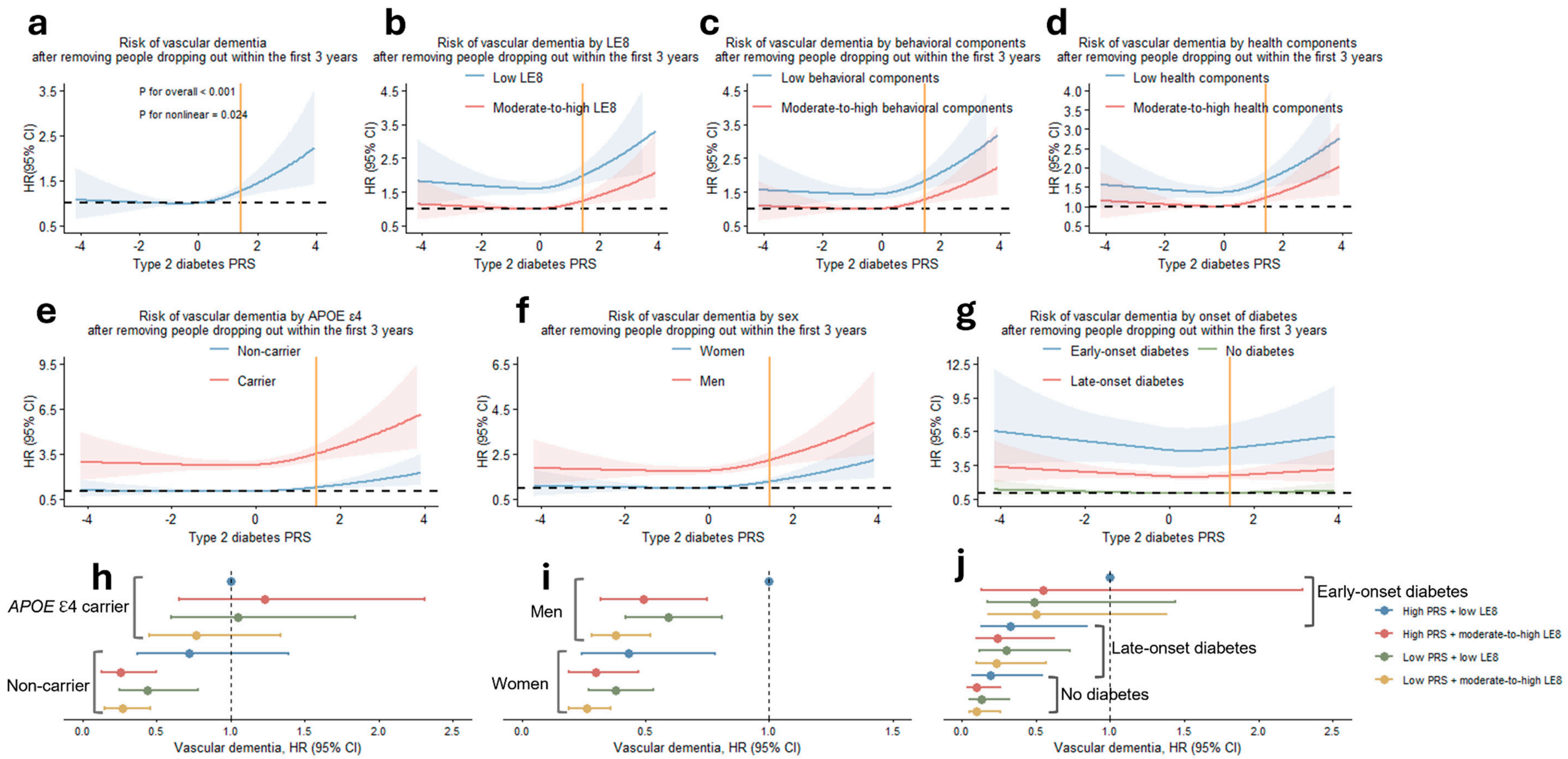

Figure S11. Association between polygenic risk score for type 2 diabetes and risk of vascular dementia in the three-year landmark analysis

Note: The pink vertical line in the graph indicates the 95% percentile of polygenic risk score for type 2 diabetes (1.45).

Abbreviations: HR: hazards ratio; CI: confidence interval; PRS: polygenic risk score; LE8: Life’s essential 8; VAD: vascular dementia.

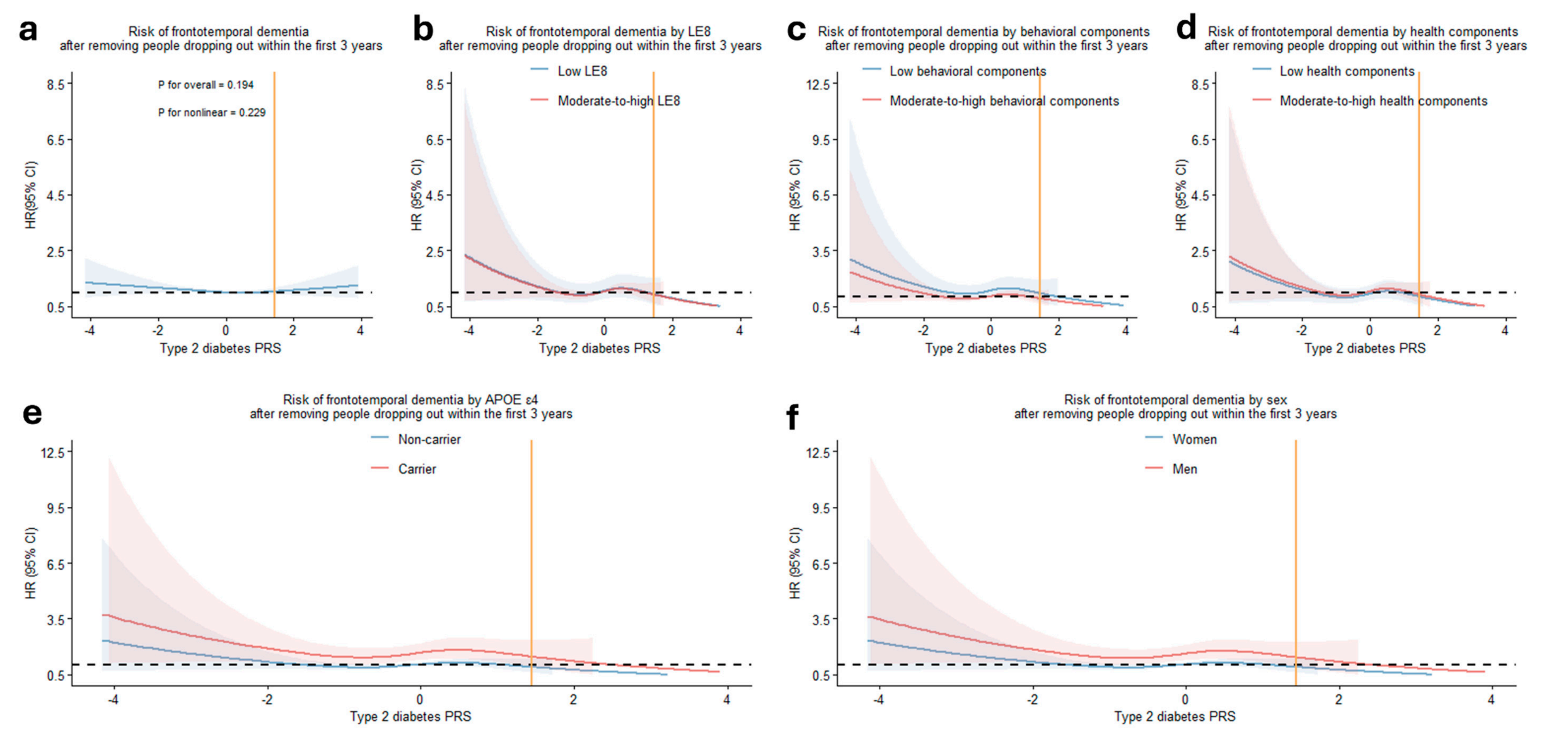

Figure S12. Association between polygenic risk score for type 2 diabetes and risk of frontotemporal dementia in the three-year landmark analysis

Note: The pink vertical line in the graph indicates the 95% percentile of polygenic risk score for type 2 diabetes (1.45).

Abbreviations: HR: hazards ratio; CI: confidence interval; PRS: polygenic risk score; LE8: Life's essential 8.

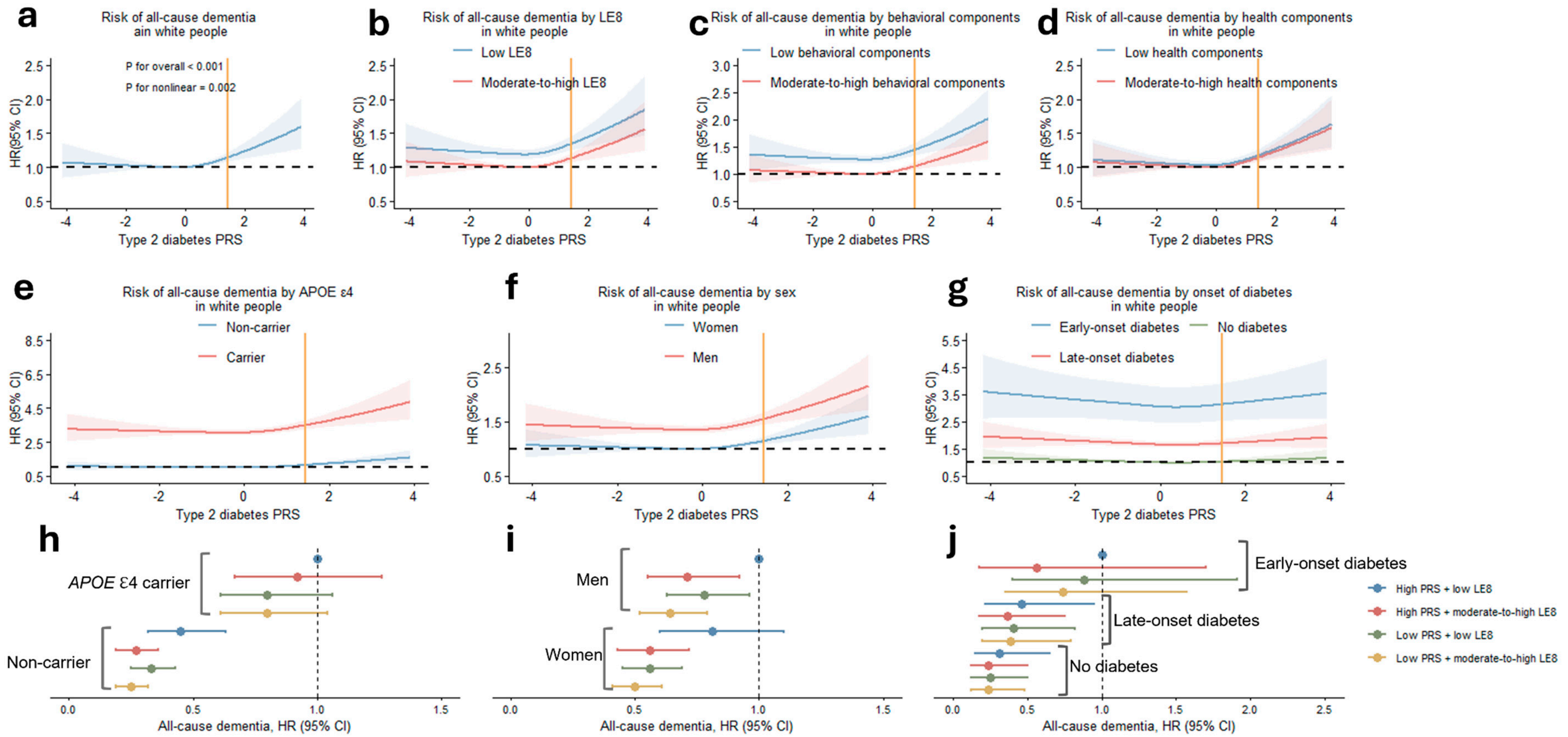

Figure S13. Association between polygenic risk score for type 2 diabetes and risk of all-cause dementia in people of White ethnic background

Note: The pink vertical line in the graph indicates the 95% percentile of polygenic risk score for type 2 diabetes (1.45).

Abbreviations: HR: hazards ratio; CI: confidence interval; PRS: polygenic risk score; LE8: Life's essential 8.

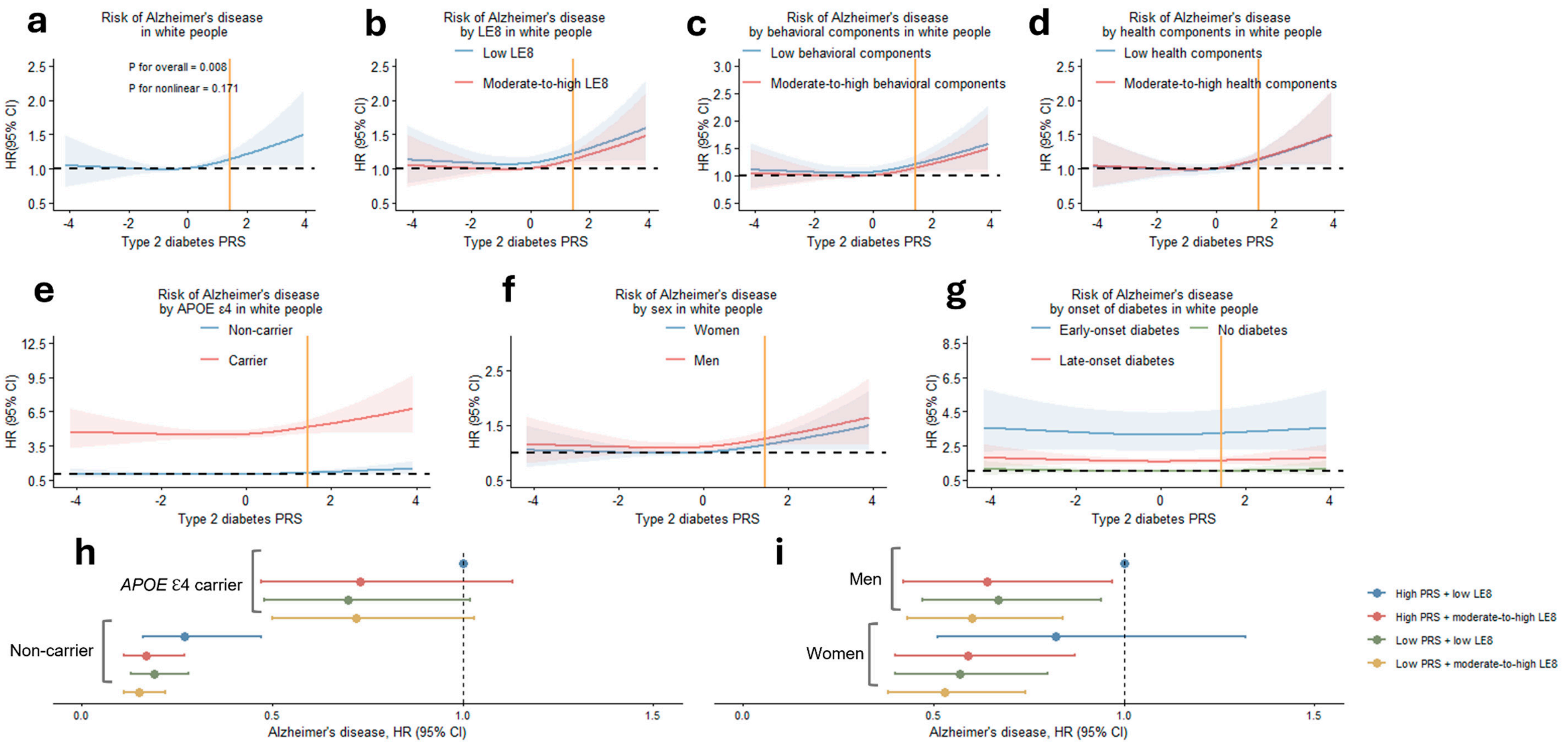

Figure S14. Association between polygenic risk score for type 2 diabetes and risk of Alzheimer's disease in people of White ethnic background

Note: The pink vertical line in the graph indicates the 95% percentile of polygenic risk score for type 2 diabetes (1.45).

Abbreviations: HR: hazards ratio; CI: confidence interval; PRS: polygenic risk score; LE8: Life's essential 8; AD: Alzheimer's disease.

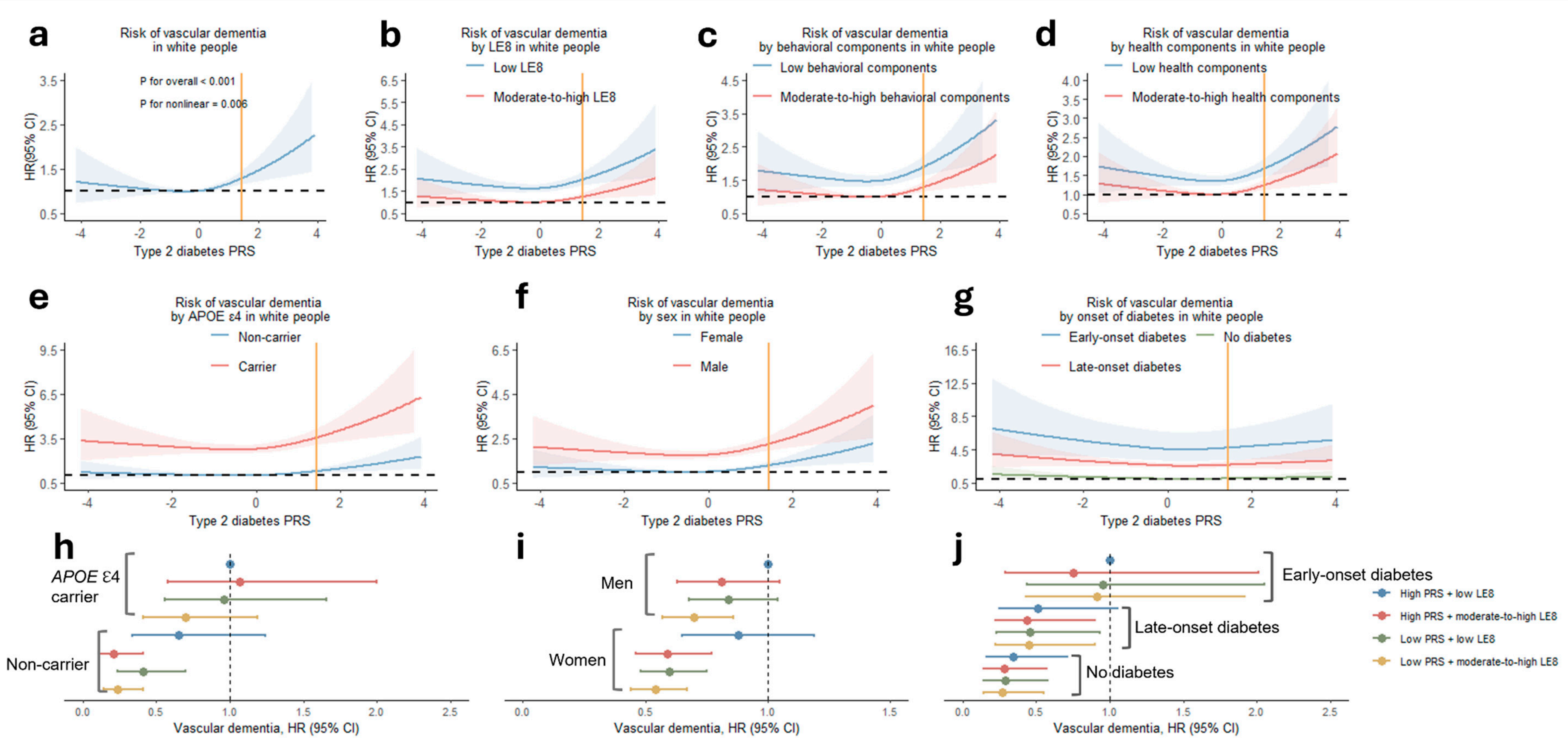

Figure S15. Association between polygenic risk score for type 2 diabetes and risk of vascular dementia in people of White ethnic background

Note: The pink vertical line in the graph indicates the 95% percentile of polygenic risk score for type 2 diabetes (1.45).

Abbreviations: HR: hazards ratio; CI: confidence interval; PRS: polygenic risk score; VAD: vascular dementia.

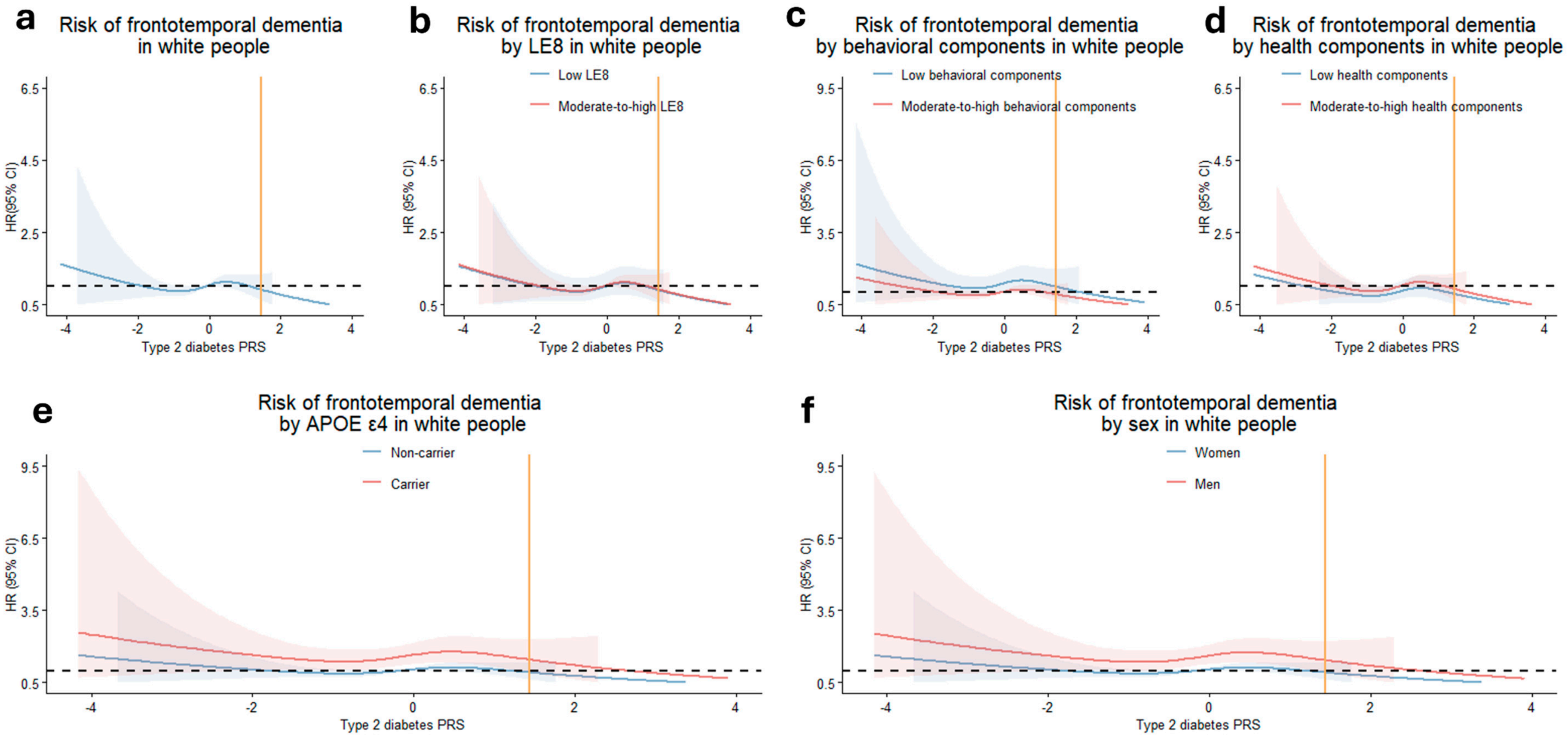

Figure S16. Association between polygenic risk score for type 2 diabetes and risk of frontotemporal dementia in people of White ethnic background

Note: The pink vertical line in the graph indicates the 95% percentile of polygenic risk score for type 2 diabetes (1.45).

Abbreviations: HR: hazards ratio; CI: confidence interval; PRS: polygenic risk score; LE8: Life's essential 8.

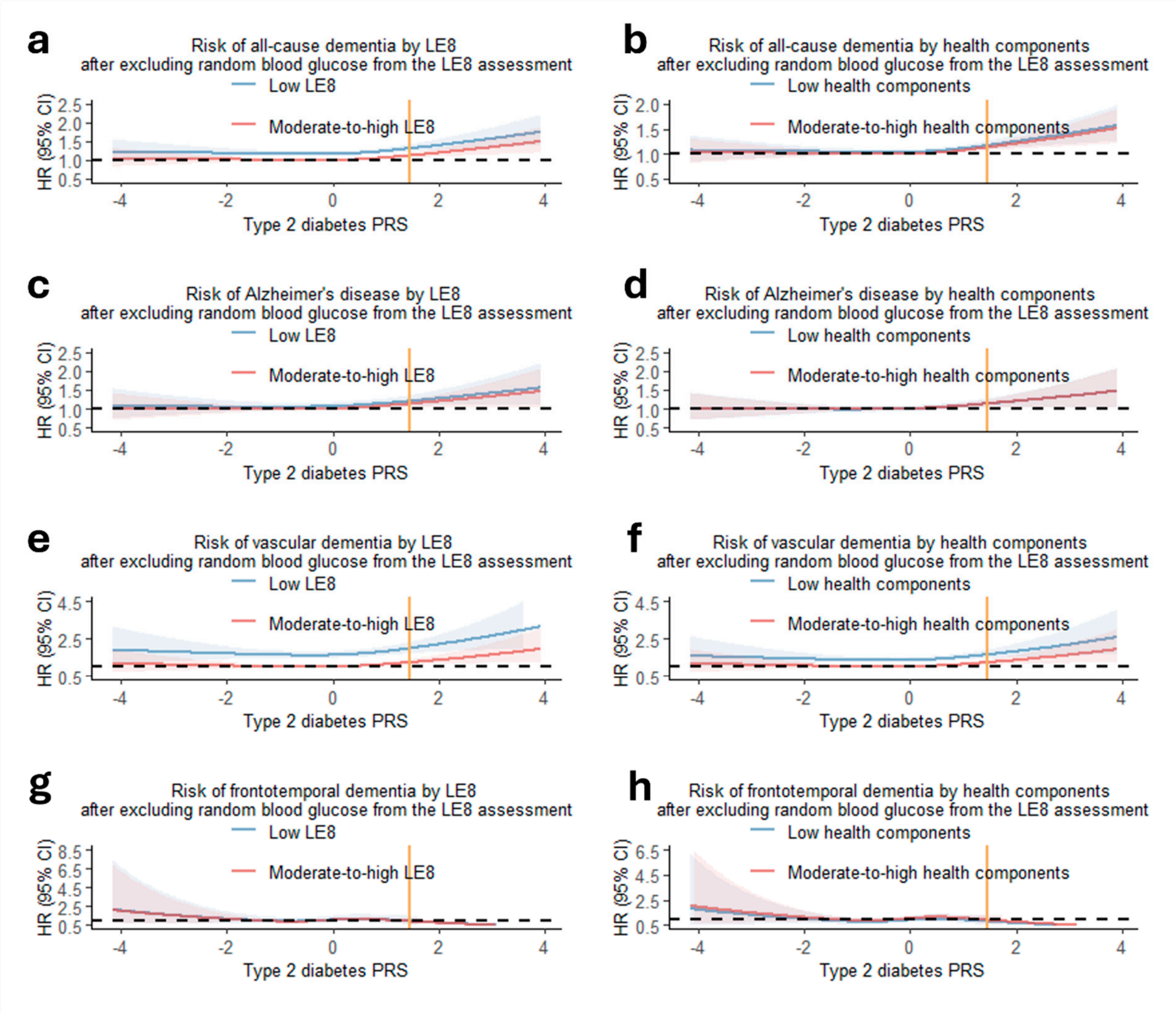

Figure S17. Association between polygenic risk score for type 2 diabetes and dementia by LE8 after excluding random blood glucose from LE8 assessment

Note: The pink vertical line in the graph indicates the 95% percentile of polygenic risk score for type 2 diabetes (1.45).

Abbreviations: HR: hazards ratio; CI: confidence interval; PRS: polygenic risk score; LE8: Life's essential 8.

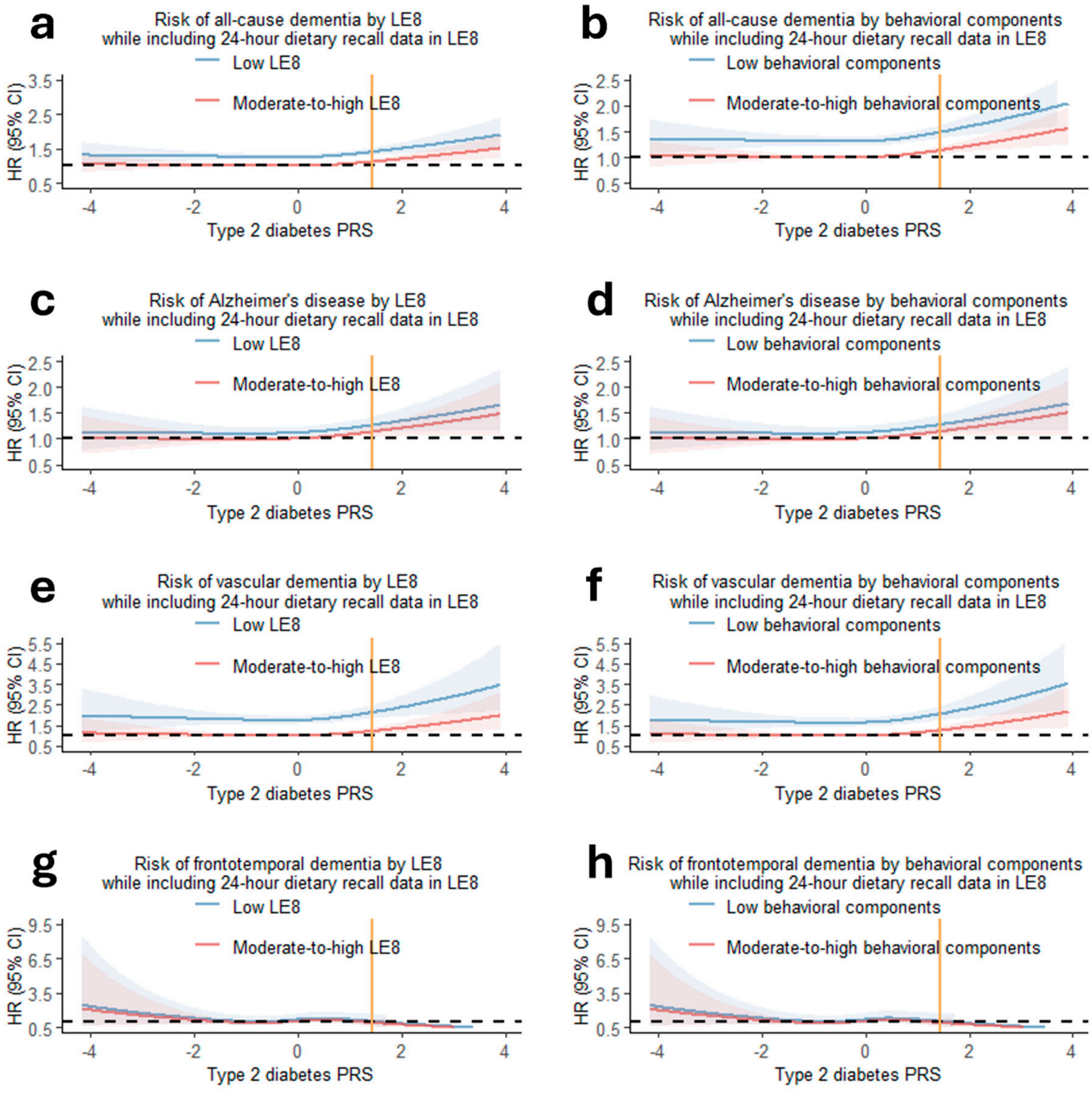

Figure S18. Association between polygenic risk score for type 2 diabetes and dementia by LE8 when including 24-hour dietary recall data in LE8 assessment

Note: The pink vertical line in the graph indicates the 95% percentile of polygenic risk score for type 2 diabetes (1.45).

Abbreviations: HR: hazards ratio; CI: confidence interval; PRS: polygenic risk score; LE8: Life's essential 8.
